# Trends and projections in cause-specific mortality among patients with colorectal cancer in the United States, 1999–2040

**DOI:** 10.3389/fpubh.2026.1850707

**Published:** 2026-08-25

**Authors:** Ziyan He, Yadong Chen, Yuxing Hu, Jiajia Lin, Yifan Wang, Caiqiu Deng, Yue Cong, Jiaming Liu, Rui Zhou, Yihan He, Fangfang Hou, Haibo Zhang

**Affiliations:** 1 The Second Clinical College of Guangzhou University of Chinese Medicine, Guangzhou, China; 2 Department of Oncology, Guangdong Provincial Hospital of Chinese Medicine, Guangzhou, China; 3 Guangdong Provincial Key Laboratory of Clinical Research on Traditional Chinese Medicine Syndrome, Guangzhou, China; 4 State Key Laboratory of Traditional Chinese Medicine Syndrome, Guangzhou, China; 5 State Key Laboratory of Dampness Syndrome of Chinese Medicine, The Second Affiliated Hospital of Guangzhou University of Chinese Medicine, Guangzhou, China

**Keywords:** CDC WONDER, colorectal cancer, health disparities, joinpoint regression, mortality trends, multiple cause of death

## Abstract

**Background:**

As CRC survival improves, the shifting contributions of metastatic and non-cancer causes to mortality remain poorly characterized, with implications for survivorship care and public health policy. This study aimed to examine temporal trends in CRC mortality with selected contributing conditions from 1999 to 2023 and project rates through 2040 across demographic and geographic subgroups in the United States.

**Methods:**

This population-based cohort study utilized mortality data from the Centers for Disease Control and Prevention Wide-ranging Online Data for Epidemiologic Research (CDC WONDER) database. The study included 1,323,609 US residents aged 25 years or older who died from 1999 through 2023 with colorectal cancer listed as the underlying cause of death. We analyzed selected contributing conditions recorded in the multiple cause-of-death field, including heart failure, ischemic heart disease, liver metastasis, lung metastasis, pulmonary embolism, and sepsis. Age-adjusted mortality rates (AAMRs) were calculated per 100,000 persons. Temporal trends were analyzed using Joinpoint regression to estimate annual percent change (APC) and average annual percent change (AAPC). Future mortality rates were projected to 2040 using autoregressive integrated moving average (ARIMA) modeling.

**Results:**

Overall CRC AAMR declined from 32.06 in 1999 to 19.57 in 2023 (AAPC,−2.08%). Recent significant increases were observed in liver metastasis mortality from 2016 to 2023 (APC: 2.89%), lung metastasis mortality from 2017 to 2023 (APC: 2.08%), pulmonary embolism mortality from 2012 to 2023 (APC: 3.59%), and heart failure mortality from 2013 to 2023 (APC: 3.16%). These trends were most pronounced among adults aged 35–64 years, non-Hispanic Black individuals, and residents of the Southern US. Model-based projections indicate that overall lung metastasis mortality will continue to increase through 2040, whereas liver metastasis mortality will peak in 2028 and subsequently decline. In pre-pandemic sensitivity analyses, the post-2012 increase in PE mortality persisted, whereas recent increases in IHD, HF, liver metastasis, and sepsis mortality were attenuated or no longer statistically significant.

**Conclusions:**

Despite declining overall CRC mortality, recent increases were observed in several metastatic and noncancer contributing conditions. However, pre-pandemic sensitivity analyses suggested that some late-period changes may have been amplified during the COVID-19 era, whereas the increase in PE mortality clearly preceded the pandemic.

## Introduction

Colorectal cancer (CRC) remains the second leading cause of cancer-related death in the United States (1), with an estimated 52,580 deaths in 2022 (2). Over the past two decades, CRC mortality has declined by over 50% since the 1970s (2, 3), driven by widespread screening, earlier diagnosis, and advances in surgical and systemic therapies (4–11). As a result, the population of CRC survivors has grown substantially, shifting the focus toward long-term complications and comorbidities that now contribute significantly to mortality (12, 13). Notably, non-cancer-related causes, including cardiovascular disease (CVD) (14, 15), thromboembolic events (16, 17), and infections (18, 19) as well as metastatic complications (20–25), may increasingly shape the mortality landscape among individuals with CRC.

While prior studies have documented declining overall CRC mortality, the evolving patterns of cause-specific death remain poorly characterized (26–28). Emerging evidence suggests a rise in pulmonary embolism (PE)-related mortality among cancer patients (29, 30), and metastatic disease continues to dominate late-stage CRC outcomes (31, 32). However, comprehensive, nationally representative data on the shifting contributions of cardiovascular, metastatic, thromboembolic, and infectious causes to CRC mortality are lacking. Furthermore, it is unknown whether these trends vary meaningfully by age (particularly among younger adults), sex, race and ethnicity, or geography-factors that may reflect disparities in screening access, treatment quality, and risk factor profiles.

To address these gaps, we analyzed data from the Centers for Disease Control and Prevention Wide-ranging Online Data for Epidemiologic Research (CDC WONDER) database (33) to assess temporal trends in cause-specific mortality among US adults with CRC from 1999 to 2023. We further projected mortality rates through 2040 and examined disparities across key demographic and geographic strata, including age, sex, race and ethnicity, US census region, and urbanization.

## Methods

### Study design and database

This population-based cohort study used data from the publicly available CDC WONDER database (33), derived from the National Vital Statistics System (NVSS) Multiple Cause of Death files (34, 35). The NVSS collects and compiles death certificate information from all 50 US states and the District of Columbia, representing more than 99% of deaths among US residents. Data were retrieved for the years 1999 through 2023. Because all data are deidentified and publicly available, institutional review board approval and informed consent were waived in accordance with the Common Rule. This study followed the Strengthening the Reporting of Observational Studies in Epidemiology (STROBE) reporting guideline (36).

### Study population and case definitions

We identified deaths among US adults aged 25 years or older from January 1, 1999, to December 31, 2023, with CRC listed as the underlying cause of death (ICD-10 codes C18-C20) (37). The lower age cutoff of 25 years was prespecified because annual deaths involving CRC and the selected contributing conditions were sparse among persons aged 18 to 24 years and were frequently subject to CDC WONDER suppression of cells containing 0 to nine deaths. Inclusion of this age group would therefore have produced incomplete annual time series unsuitable for consistent Joinpoint and ARIMA analyses (33). To assess potential shifts in the patterns of cause-specific mortality over time, we further classified these deaths according to selected contributing conditions recorded in the multiple cause-of-death field: ischemic heart disease (I20–I25), heart failure (I50), secondary malignant neoplasms of the liver (C78.7) and lung (C78.0), pulmonary embolism (I26), and sepsis (A40–A41), based on WHO and NCHS ICD-10 mortality coding guidance (37–40). Each selected condition was assessed independently. The categories were not mutually exclusive, and a decedent could be included in more than one condition-specific analysis if multiple selected conditions were recorded on the death certificate. Throughout this study, mortality with a selected contributing condition refers to deaths in which CRC was recorded as the underlying cause and the selected condition was recorded anywhere in the multiple cause-of-death field. These estimates do not represent competing-risk or condition-specific mortality in a longitudinal cohort of patients with CRC. Pulmonary embolism and sepsis were selected as representative thromboembolic and severe infectious complications, respectively (30, 41). This comprehensive classification enabled us to evaluate whether non-cancer-related comorbidities and metastatic complications have become increasingly prominent contributors to death among individuals with CRC in the context of improving overall cancer survival.

### Data stratification and covariates

Demographic and geographic variables extracted from the CDC WONDER database included age (categorized into 10-year groups from 25 to 85+), sex (male, female), race and ethnicity (Hispanic, non-Hispanic White, non-Hispanic Black, non-Hispanic Asian, and non-Hispanic American Indian or Alaska Native), census region (Northeast, Midwest, South, and West), and urbanization category (large metropolitan, medium or small metropolitan, and nonmetropolitan areas) based on the 2013 National Center for Health Statistics Urban-Rural Classification Scheme. Analyses involving urbanization were restricted to 1999–2020 due to unavailability of urbanization data after 2020.

### Statistical analysis

Crude mortality rates (CMRs) were calculated as the annual number of CRC-related deaths divided by the corresponding US population. Age-adjusted mortality rates (AAMRs) per 100,000 persons were derived using direct standardization to the 2000 US Standard Population (42, 43). Temporal trends in AAMRs from 1999 to 2023 were analyzed with Joinpoint Regression Software (version 5.4.0; National Cancer Institute), which identifies significant inflection points (“joinpoints”) by fitting joined log-linear segments and estimates annual percent change (APC) and average annual percent change (AAPC) with 95% confidence intervals (CIs). Up to four joinpoints were permitted based on time points and model fit (44–46). APCs and AAPCs were considered statistically significant (increasing or decreasing) if the slope differed from zero (*P* ≤ 0.05, 2-tailed *t* test). To ensure consistent reporting, AAPCs were used exclusively to summarize the net temporal change over the entire study period, whereas APCs were used to describe trends within joinpoint-defined segments. For cross-outcome comparisons of recent trends, we consistently reported the APC for the final joinpoint-defined segment, regardless of its magnitude or statistical significance. Earlier segments were reported only when necessary to describe changes in slope or direction, and AAPCs and APCs were not compared directly as measures of relative trend magnitude.

To assess the potential influence of the COVID-19 pandemic on the observed late-period trends, we performed a sensitivity analysis restricting the Joinpoint regression analyses to the pre-pandemic period from 1999 through 2019. The same model-selection parameters and statistical significance criteria as those used in the primary analysis were applied. The analyses were repeated for heart failure, ischemic heart disease, liver metastasis, lung metastasis, pulmonary embolism, and sepsis in the overall population.

To forecast mortality rates through 2040, we fitted nonseasonal autoregressive integrated moving average (ARIMA) models (47–49) to annual mortality rates observed from 1999 to 2023 using Python version 3.13.5 (Python Software Foundation, Wilmington, DE, USA) and StatsForecast version 2.0.3 (Nixtla, Inc., San Francisco, CA, USA). For each series, the ARIMA (*p, d, q*) order and the inclusion of a mean or drift term were selected by stepwise minimization of the corrected Akaike information criterion (AICc), with nonseasonal differencing determined using the Kwiatkowski-Phillips-Schmidt-Shin procedure. The maximum values of *p* and *q* were 5, the maximum value of d was 2, and *p* + *q* was restricted to 5 or less. If the initial automatically selected model showed residual autocorrelation (Ljung-Box *P* < 0.05) or generated any negative point forecast from 2024 to 2040, candidate models with the same differencing order were evaluated, and the lowest-AICc model satisfying both criteria was retained. Residual autocorrelation was assessed using the Ljung-Box test at lag 10, with degrees of freedom calculated as 10 – (*p* + *q*). Forecast accuracy was evaluated using 10 rolling-origin, one-step-ahead cross-validation folds, with the selected model specification refitted at each forecast origin and RMSE (47) calculated across the 10 folds. Forecasts were generated through 2040 with 95% prediction intervals. Model specifications and diagnostic results are reported in Supplementary Table S4, and annual forecasts are presented in Supplementary Table S5. In the figures, prediction-interval shading was displayed only through the last year in which the lower prediction limit remained nonnegative, whereas point forecasts were displayed through 2040.

## Results

### Overall population

**Figure 1:**
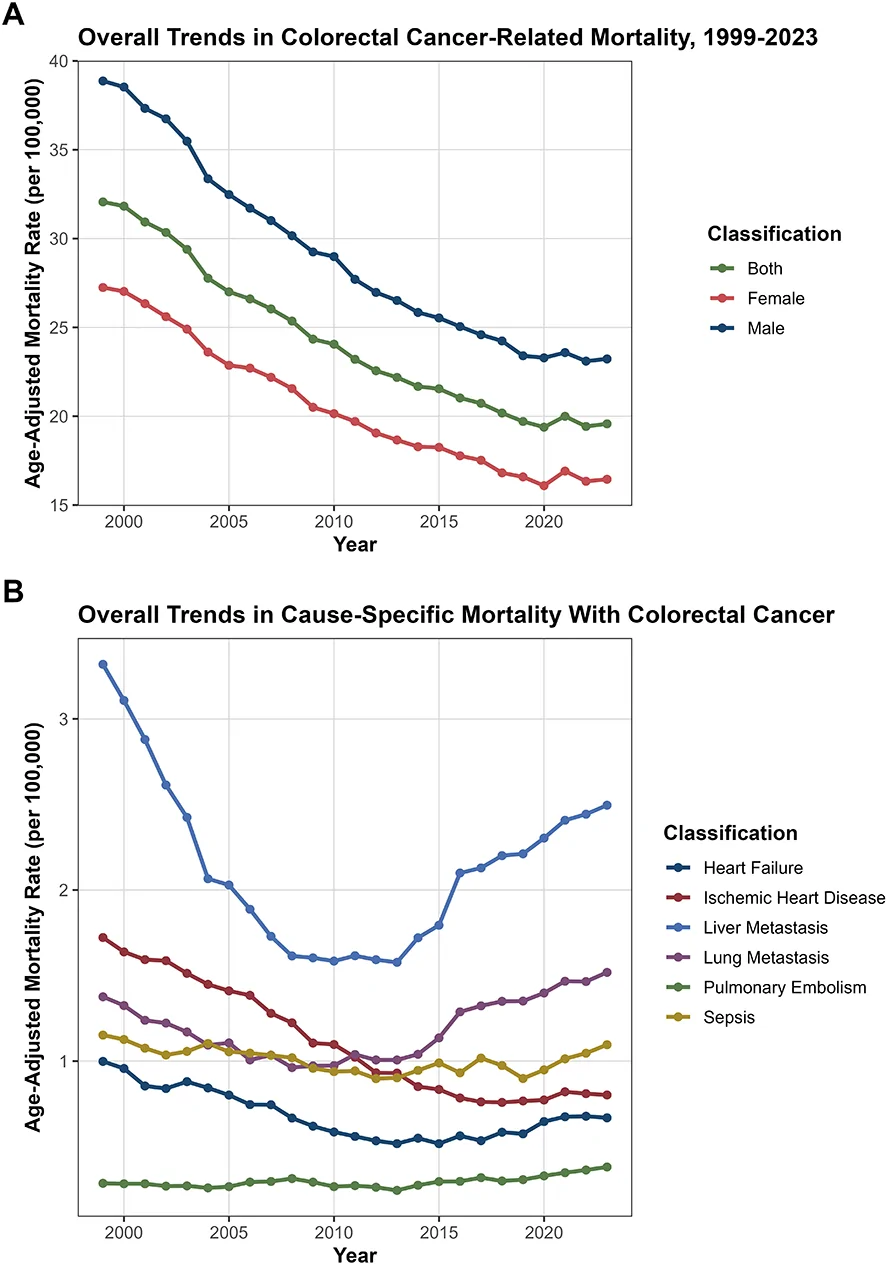
Trends in age-adjusted mortality rates for colorectal cancer and major contributing causes among US Adults, 1999–2023. **(A)** Overall trends in colorectal cancer-related mortality, 1999–2023. **(B)** Overall trends in cause-specific mortality with colorectal cancer.

**Figure 2:**
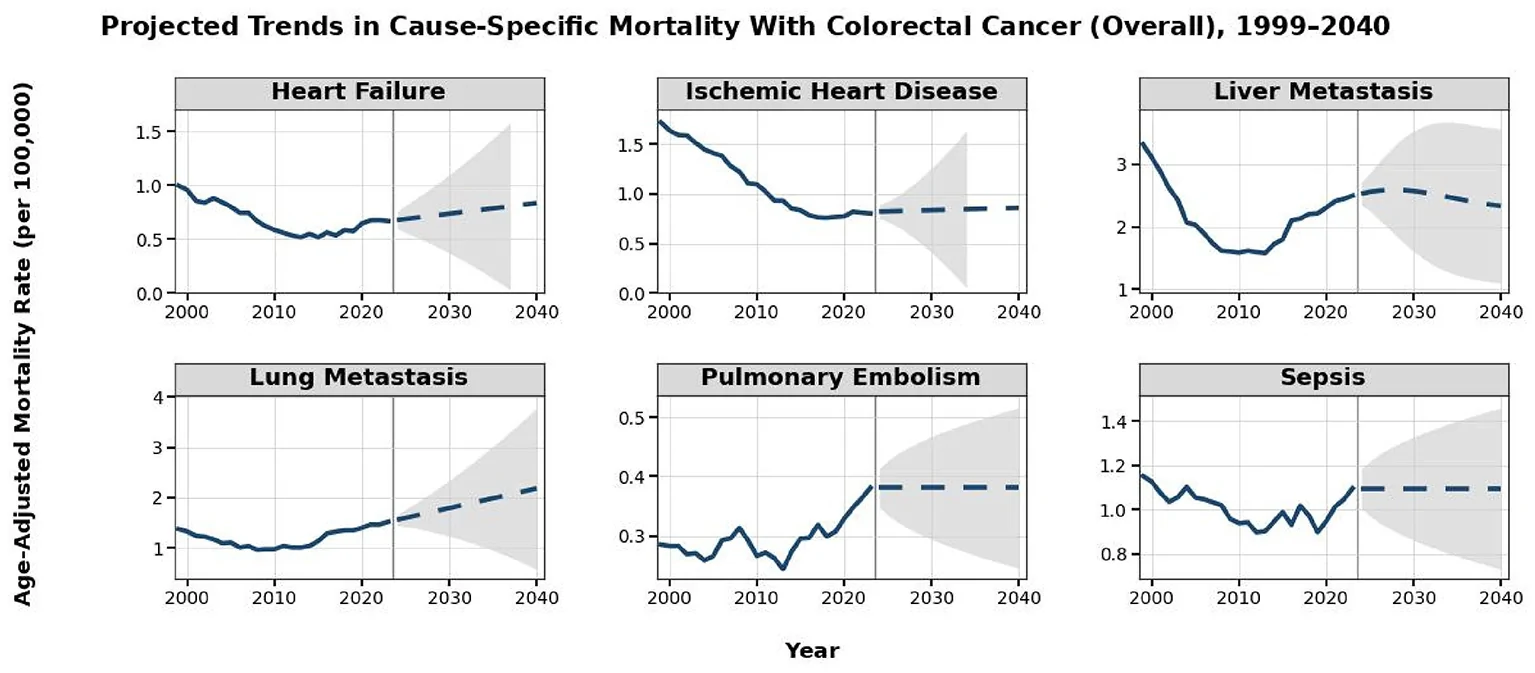
Age-adjusted mortality rates and projections for cause-specific mortality among patients with colorectal cancer, United States, 1999–2040. Solid blue lines represent observed mortality rates from 1999 to 2023; dashed blue lines represent model-based forecasts from 2024 to 2040. Shaded gray areas indicate 95% prediction intervals and are displayed only through the last year in which the lower prediction limit remains nonnegative.

From 1999 to 2023, 1 323 609 deaths with colorectal cancer (CRC) as the underlying cause were recorded in the United States. The AAMR declined from 32.06 in 1999 to 19.57 in 2023 (AAPC, −2.08%), with a steeper decrease from 1999 to 2012, a slower decline from 2012 to 2020, and a plateau thereafter. The annual number of CRC deaths fell from 56,642 in 1999 to 51,075 in 2012 but rose again to 53,456 in 2023 (Figure 1A and Table 1). State-level trends are shown in Supplementary Figure S1. Ischemic heart disease (IHD)-related mortality exhibited an overall decline (AAPC: −3.02%, *P* < 0.001); however, this trajectory was not strictly continuous. A statistically significant reversal occurred at the overall population level from 2017 to 2023 (APC: 1.64%, *P* = 0.001). Similarly, heart failure (HF)-related mortality reversed from an initial decline to a significant increase after 2013. Mortality from liver metastasis followed a U-shaped pattern, reaching its lowest AAMR in 2013 and rising to 2.50 in 2023, and lung metastasis-related deaths in 2023 exceeded those in 1999. Pulmonary embolism (PE)-related mortality exhibited a nonmonotonic trajectory prior to 2012, characterized by alternating periods of decline and growth (APC 1999 to 2004: −1.95%, *P* = 0.059; APC 2004 to 2008: 4.26%, *P* = 0.032; APC 2008 to 2012: −4.86%, *P* = 0.032). Subsequently, the trend entered a phase of sustained increase (APC 2012 to 2023: 3.59%, *P* = 0.003). This shifting pattern resulted in the overall AAMR for PE rising from 0.29 in 1999 to 0.38 in 2023, representing an increase of 31%. Furthermore, sepsis mortality rebounded after 2012 despite demonstrating a modest overall decline (Figure 1B, Table 1, Supplementary Tables S1, S2). Projections indicate that overall CRC AAMR will decline to 17.66 by 2040. Liver metastasis-related AAMR is projected to peak at 2.59 in 2028 and subsequently decline to 2.33 by 2040, whereas lung metastasis-related AAMR is projected to increase to 2.18 by 2040 (Figure 2 and Supplementary Table S5).

**Table 1:** Observed trends in age-adjusted mortality with colorectal cancer and metastatic causes, 1999–2023.

Characteristic	Colorectal cancer			Liver metastasis			Lung metastasis		
	AAMR (95% CI)		AAPC, *P* value	AAMR (95% CI)		AAPC, *P* value	AAMR (95% CI)		AAPC, *P* value
	1999	2023		1999	2023		1999	2023	
Sex									
Both	32.06 (31.80–32.33)	19.57 (19.40–19.74)	−2.08 (−2.22 to −1.99), *P* < 0.001	3.32 (3.23–3.40)	2.50 (2.43–2.56)	−1.17 (−1.34 to −1.02), *P* < 0.001	1.38 (1.32–1.43)	1.52 (1.47–1.57)	0.41 (0.21 to 0.58), *P* < 0.001
Female	27.25 (26.93–27.56)	16.45 (16.24–16.66)	−2.12 (−2.31 to −1.99), *P* < 0.001	2.72 (2.62–2.82)	1.95 (1.88–2.03)	−1.23 (−1.42 to −1.06), *P* < 0.001	1.03 (0.97–1.09)	1.19 (1.13–1.24)	0.51 (0.19 to 0.80), *P* = 0.003
Male	38.87 (38.41–39.33)	23.23 (22.95–23.50)	−2.15 (−2.23 to −2.06), *P* < 0.001	4.15 (4.00–4.30)	3.12 (3.02–3.22)	−1.29 (−1.45 to −1.14), *P* < 0.001	1.88 (1.78–1.98)	1.86 (1.78–1.93)	−0.00 (−0.13 to 0.13), *P* = 0.940
Race									
Hispanic	21.98 (20.97–22.99)	16.08 (15.62–16.54)	−1.33 (−1.51 to −1.19), *P* < 0.001	2.25 (1.93–2.56)	2.06 (1.90–2.22)	−0.33 (−0.96 to 0.53), *P* = 0.352	1.10 (0.87–1.32)	1.24 (1.11–1.37)	0.75 (0.03 to 1.83), *P* = 0.040
NH American Indian	22.72 (19.03–26.42)	20.12 (17.96–22.27)	−0.92 (−1.36 to −0.40), *P* < 0.001	NA	2.37 (1.70–3.23)	2.06 (−0.62 to 5.15), *P* = 0.118	NA	NA	NA
NH Asian	18.54 (17.20–19.89)	13.34 (12.76–13.92)	−1.68 (−1.95 to −1.35), *P* < 0.001	2.07 (1.65–2.57)	1.71 (1.50–1.92)	−0.70 (−1.43 to 0.19), *P* = 0.122	1.00 (0.72–1.35)	1.27 (1.09–1.45)	0.86 (−0.02 to 2.17), *P* = 0.059
NH Black	44.00 (42.93–45.07)	24.90 (24.30–25.50)	−2.49 (−2.69 to −2.35), *P* < 0.001	4.85 (4.50–5.21)	3.07 (2.86–3.28)	−1.92 (−2.30 to −1.52), *P* < 0.001	1.75 (1.54–1.96)	1.65 (1.50–1.81)	−0.13 (−0.68 to 0.53), *P* = 0.641
NH White	31.72 (31.44–32.01)	20.04 (19.83–20.24)	−1.94 (−2.07 to −1.85), *P* < 0.001	3.24 (3.15–3.34)	2.58 (2.51–2.66)	−0.99 (−1.12 to −0.88), *P* < 0.001	1.33 (1.27–1.39)	1.59 (1.53–1.65)	0.59 (0.38 to 0.77), *P* < 0.001
Age group									
25–34 years	0.69 (0.61–0.77)	0.81 (0.72–0.89)	0.45 (−0.01 to 0.95), *P* = 0.052	0.07 (0.05–0.10)	0.14 (0.10–0.17)	NA	NA	NA	NA
35–44 years	2.87 (2.72–3.03)	3.71 (3.53–3.88)	1.03 (0.85 to 1.21), *P* < 0.001	0.29 (0.24–0.34)	0.62 (0.54–0.69)	3.26 (2.71 to 3.88), *P* < 0.001	0.12 (0.09–0.15)	0.35 (0.30–0.41)	5.23 (4.38 to 6.35), *P* < 0.001
45–54 years	10.88 (10.55–11.22)	12.14 (11.80–12.48)	0.45 (0.28 to 0.68), *P* = 0.006	1.26 (1.15–1.38)	1.98 (1.84–2.12)	1.66 (1.11 to 2.14), *P* < 0.001	0.48 (0.41–0.55)	1.20 (1.09–1.30)	3.67 (3.09 to 4.35), *P* < 0.001
55–64 years	33.17 (32.43–33.90)	24.26 (23.79–24.73)	−1.43 (−1.58 to −1.26), *P* < 0.001	3.99 (3.73–4.24)	3.71 (3.52–3.89)	−0.41 (−0.73 to −0.09), *P* = 0.014	1.59 (1.43–1.75)	2.18 (2.04–2.32)	1.67 (1.18 to 2.28), *P* < 0.001
65–74 years	76.81 (75.54–78.07)	39.84 (39.18–40.51)	−2.69 (−2.80 to −2.57), *P* < 0.001	9.34 (8.90–9.78)	5.32 (5.08–5.56)	−2.37 (−2.65 to −2.13), *P* < 0.001	3.76 (3.48–4.04)	3.28 (3.09–3.47)	−0.50 (−0.89 to −0.15), *P* = 0.025
75–84 years	145.84 (143.70–147.98)	69.63 (68.42–70.83)	−3.20 (−3.29 to −3.12), *P* < 0.001	15.09 (14.40–15.78)	8.08 (7.67–8.49)	−2.36 (−2.63 to −2.09), *P* < 0.001	6.54 (6.09–7.00)	4.99 (4.67–5.32)	−1.13 (−1.47 to −0.80), *P* < 0.001
85+ years	270.27 (265.27–275.27)	157.66 (154.53–160.79)	−2.35 (−2.53 to −2.18), *P* < 0.001	17.26 (16.00–18.52)	12.69 (11.80–13.57)	−1.27 (−1.57 to −0.96), *P* < 0.001	8.04 (7.18–8.90)	7.85 (7.15–8.54)	−0.19 (−0.73 to 0.38), *P* = 0.435
Census region									
Midwest	33.99 (33.43–34.55)	20.13 (19.75–20.50)	−2.22 (−2.34 to −2.12), *P* < 0.001	3.75 (3.57–3.94)	2.76 (2.62–2.90)	−1.27 (−1.64 to −0.95), *P* < 0.001	1.39 (1.27–1.50)	1.64 (1.53–1.75)	0.43 (0.06 to 0.81), *P* = 0.027
Northeast	35.03 (34.43–35.63)	17.31 (16.94–17.68)	−3.08 (−3.18 to −2.98), *P* < 0.001	3.53 (3.34–3.72)	1.43 (1.32–1.54)	−3.85 (−4.12 to −3.61), *P* < 0.001	1.38 (1.27–1.50)	0.88 (0.79–0.96)	−2.22 (−2.53 to −1.91), *P* < 0.001
South	31.33 (30.89–31.77)	21.13 (20.84–21.41)	−1.74 (−1.89 to −1.63), *P* < 0.001	3.28 (3.14–3.42)	2.88 (2.77–2.98)	−0.80 (−1.10 to −0.55), *P* < 0.001	1.41 (1.32–1.50)	1.71 (1.62–1.79)	0.80 (0.63 to 0.96), *P* < 0.001
West	27.79 (27.24–28.34)	18.31 (17.97–18.65)	−1.75 (−1.90 to −1.62), *P* < 0.001	2.57 (2.41–2.74)	2.49 (2.36–2.61)	0.16 (−0.13 to 0.44), *P* = 0.280	1.25 (1.13–1.37)	1.57 (1.46–1.67)	1.01 (0.57 to 1.46), *P* < 0.001
Urbanization^a^									
Large metro	32.31 (31.94–32.69)	18.36 (18.13–18.58)	−2.70 (−2.80 to −2.61), *P* < 0.001	3.13 (3.01–3.25)	2.03 (1.95–2.10)	−2.03 (−2.38 to −1.74), *P* < 0.001	1.27 (1.20–1.35)	1.25 (1.19–1.31)	0.04 (−0.37 to 0.50), *P* = 0.827
Medium–small metro	30.92 (30.45–31.39)	19.31 (19.00–19.62)	−2.33 (−2.46 to −2.20), *P* < 0.001	3.54 (3.38–3.70)	2.47 (2.36–2.58)	−1.55 (−1.86 to −1.22), *P* < 0.001	1.39 (1.29–1.49)	1.37 (1.29–1.46)	0.03 (−0.21 to 0.22), *P* = 0.778
Nonmetropolitan	33.25 (32.63–33.87)	23.16 (22.69–23.64)	−1.81 (−1.94 to −1.65), *P* < 0.001	3.38 (3.18–3.58)	3.01 (2.84–3.19)	−0.89 (−1.20 to −0.57), *P* < 0.001	1.59 (1.45–1.72)	1.84 (1.70–1.97)	0.44 (−0.17 to 1.10), *P* = 0.148

^a^Urbanization strata were available through 2020 only; thus 2020 AAMRs are shown in the 2023 column for these rows.

AAMR, age-adjusted mortality rate; AAPC, average annual percent change; CI, confidence interval; NH, non-Hispanic.

NA indicates that the mortality rate or AAPC could not be estimated because one or more required annual cells contained 0 to 9 deaths and were suppressed by CDC WONDER.

### Sex

**Figure 3:**
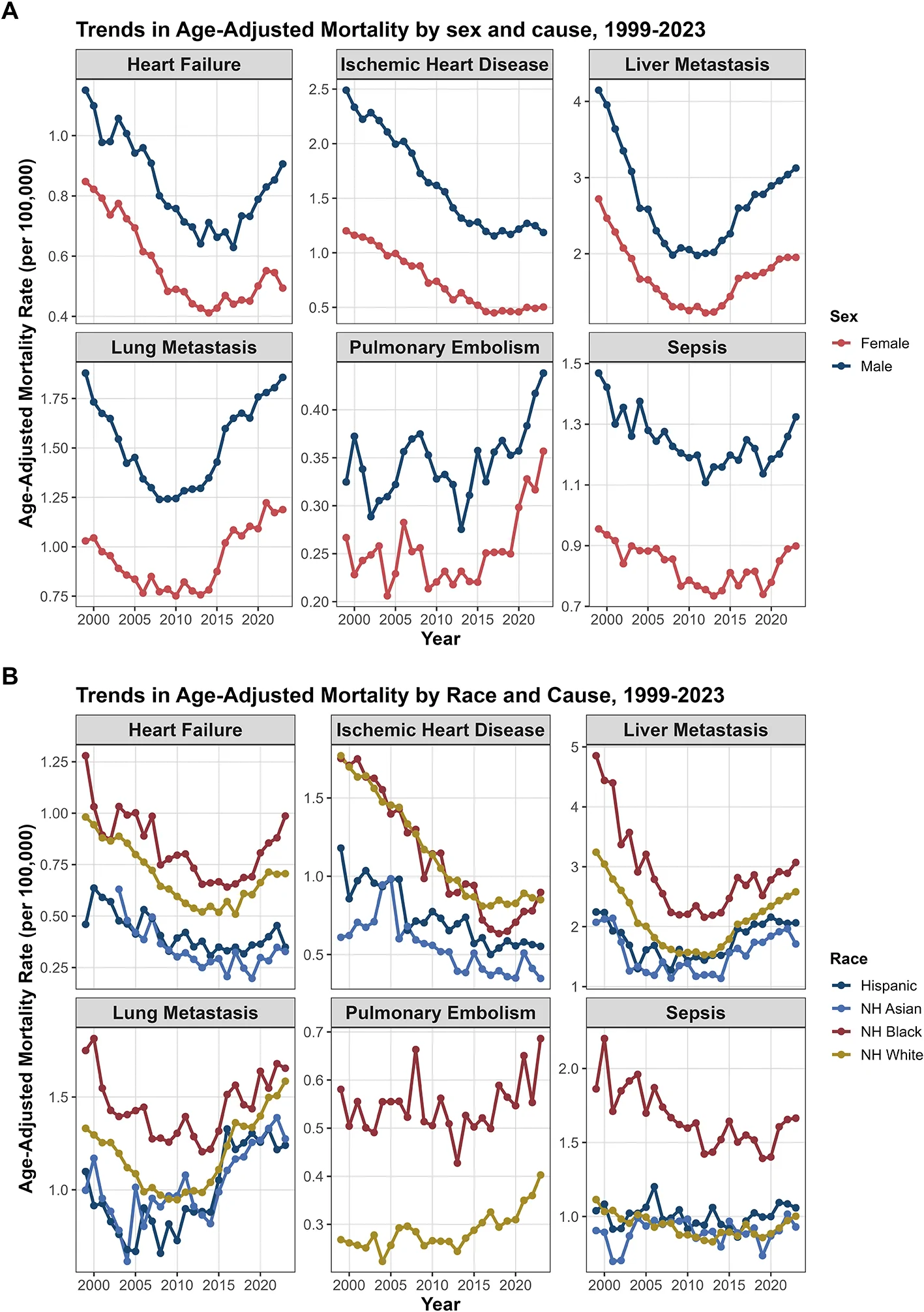
Trends in age-adjusted cause-specific mortality rates among US adults with colorectal cancer by sex and race, 1999–2023. **(A)** Trends in age-adjusted mortality by sex and cause, 1999–2023. **(B)** Trends in age-adjusted mortality by race and cause, 1999–2023.

From 1999 to 2023, men accounted for 51.8% of CRC-related deaths and had consistently higher AAMRs than women across all cause-specific categories (Figure 3A). CRC mortality declined at similar rates for both sexes (men: AAPC, −2.15%; women: −2.12%; Table 1). IHD-related mortality decreased faster in women (AAPC −3.60%) than in men (AAPC −2.81%), and forecasts suggest a modest future increase in women but continued decline in men. HF-related mortality followed a U-shaped trajectory in both sexes. Among women, mortality declined from 1999 to 2014 (APC: −4.96%, *P* < 0.001) and subsequently increased from 2014 to 2023 (APC: 3.05%, *P* < 0.001). A similar reversal was observed in men, with the recent increase beginning in 2017. PE-related mortality registered an overall significant increase in AAPC for both men (AAPC: 0.73%, *P* = 0.003) and women (AAPC: 1.34%, *P* < 0.001). Conversely, sepsis mortality experienced an overall significant decline in both men (AAPC: −0.50%, *P* < 0.001) and women (AAPC: −0.40%, *P* = 0.010). However, segment-specific annual percent change analyses documented recent significant upward trends for both conditions. Specifically, PE-related mortality increased in men from 2013 to 2023 (APC: 3.42%, *P* = 0.015) and in women from 2015 to 2023 (APC: 5.79%, *P* < 0.001). Similarly, sepsis mortality rebounded, rising in men from 2012 to 2023 (APC: 0.88%, *P* = 0.005) and in women from 2013 to 2023 (APC: 1.54%, *P* < 0.001). Notably, these recent increases were steeper in women. Furthermore, the sepsis-related AAMR in men is projected to remain approximately stable at 1.32 through 2040. For metastatic disease, liver and lung metastasis-related mortality increased in both sexes after approximately 2013, with higher AAMR in men but faster relative growth in women. Projections indicate that liver metastasis-related AAMR will generally increase in men, reaching 3.51 by 2040, whereas the rate in women will decline from 1.90 in 2024 to 1.57 in 2040. Lung metastasis-related AAMR is projected to decline in both sexes, reaching 0.99 in women and 1.52 in men by 2040 (Supplementary Figure S2 and Supplementary Table S5).

### Race and ethnicity

Non-Hispanic (NH) Black individuals had the highest AAMRs for overall CRC and most cause-specific categories throughout the study period, although CRC mortality declined most rapidly in this group (AAPC, −2.49%) compared with NH White, Hispanic, and NH Asian populations (Table 1). HF-related mortality decreased initially in both NH Black and NH White individuals but increased again after the mid-2010s, with a steeper rise and higher projected HF-related AAMRs among NH Black individuals. Although IHD-related mortality declined overall across all racial and ethnic groups, a significant recent reversal emerged among NH White individuals, with mortality increasing from 2016 to 2023 (APC: 1.12%, *P* = 0.032). Hispanic and NH Asian individuals consistently had lower AAMRs for heart failure and sepsis. Mortality from liver and lung metastases increased after 2012 across all racial and ethnic groups; by 2023, NH White, Hispanic, and NH Asian individuals all exceeded their 1999 lung metastasis-related AAMRs. Projections suggest that liver metastasis-related AAMR will remain higher among NH Black than NH White individuals. Among NH Black individuals, the projected rate will peak at 2.97 in 2025 and decline to 2.22 by 2040; among NH White individuals, it will decline from 2.58 in 2024 to 2.04 in 2040. For lung metastasis, AAMR among NH White individuals is expected to continue increasing, while that among NH Black individuals is projected to decrease modestly by 2040. Among racial and ethnic groups with estimable data, PE-related mortality increased significantly among NH White individuals; estimates were unavailable for Hispanic and NH Asian individuals because of suppressed annual death counts. Sepsis-related mortality declined slightly among NH Black individuals but remained relatively stable in other groups (Figure 3B, Table 1, and Supplementary Figure S3 and Supplementary Tables S1, S2).

### Age group

**Figure 4:**
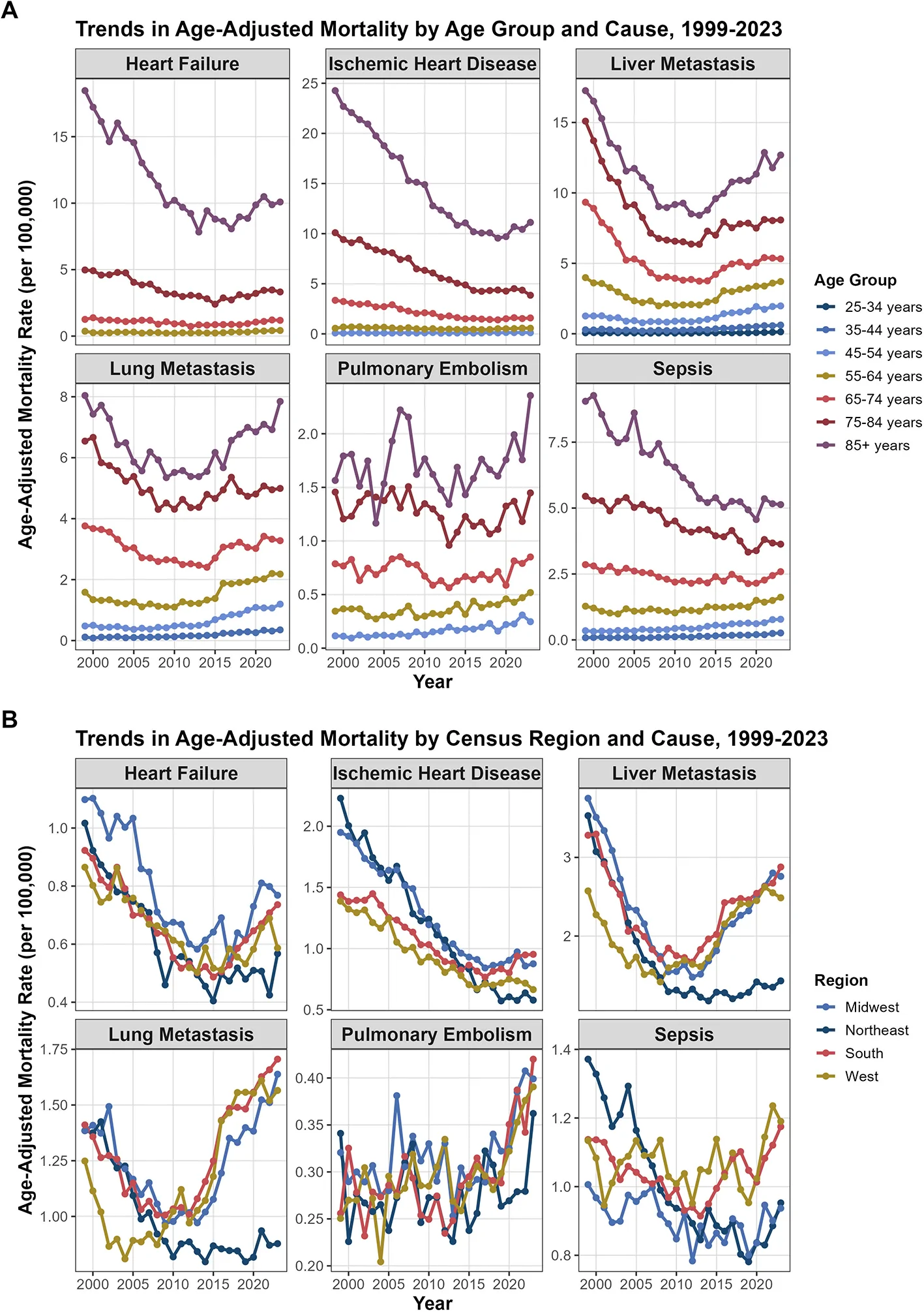
Trends in age-adjusted cause-specific mortality rates among US adults with colorectal cancer by age group and census region, 1999–2023. **(A)** Trends in age-adjusted mortality by age group and cause, 1999–2023. **(B)** Trends in age-adjusted mortality by census region and cause, 1999–2023.

Crude CRC mortality rates increased with age, with the highest burden among individuals aged 85 years or older. The crude CRC mortality rate among adults aged 55 to 64 years is projected to increase from 24.31 in 2024 to 25.20 in 2040. Metastatic CRC mortality increased among adults aged 35 to 64 years, with particularly rapid growth in liver metastasis-related mortality among adults aged 35 to 44 years and 45 to 54 years. Continued increases are projected through 2040 in these two age groups, whereas liver metastasis-related mortality among adults aged 55 to 64 years is projected to decline. In older adults, liver metastasis-related mortality is also expected to rise, with higher projected crude rates in each successive older age category. Lung metastasis-related mortality increased steeply in younger and middle-aged adults and is projected to continue increasing among adults aged 45 to 54 years, reaching 2.18 by 2040. In contrast, the projected rate among adults aged 55 to 64 years remains approximately stable at 2.18 through 2040 (Figure 4A, Supplementary Figures S4, S5, and Supplementary Table S5).

In contrast, mortality from nonmetastatic causes generally declined in individuals aged 65 years or older. HF-related mortality showed the largest declines in the oldest age groups but are projected to level off or rise slightly again, particularly among those aged 85 years or older. While IHD-related mortality demonstrated large overall declines in older populations, significant recent reversals occurred among adults aged 55 to 64 years (APC 2017 to 2023: 5.55%, *P* = 0.002) and those aged 85 years or older (APC 2019 to 2023: 3.56%, *P* = 0.003). PE-related mortality increased mainly in middle-aged adults, with stable or decreasing trends in older age groups. Notably, sepsis-related mortality among adults aged 65 to 74 years increased sharply from 2020 to 2023 (APC: 7.08%, *P* = 0.001; Supplementary Figures S4–S6).

### Census region

CRC-related mortality declined in all US census regions, with the Northeast showing the greatest reductions in overall CRC and IHD-related mortality. Despite overall reductions across all census regions, a statistically significant recent reversal in IHD mortality was documented in the South, where rates increased from 2017 to 2023 (APC: 4.26%, *P* = 0.005). By 2023, the Northeast had the lowest AAMRs for most cause-specific categories, whereas the South and Midwest had consistently higher burdens. Metastatic patterns varied across census regions. The South and the Midwest recorded recent statistically significant increases in both liver and lung metastasis-related mortality. The West demonstrated significant upward trends in these categories from 2014 to 2017. Subsequent mortality trends in the West lacked statistical significance from 2017 to 2023. Across these three regions, lung metastasis AAMRs in 2023 exceeded their 1999 baseline levels. Specifically, Joinpoint analysis revealed distinct periods of significantly increasing liver metastasis mortality in the Midwest from 2016 to 2023 (APC: 4.62%, *P* = 0.030) and in the South from 2012 to 2016 (APC: 8.48%, *P* = 0.010) and from 2016 to 2023 (APC: 2.62%, *P* = 0.023). In contrast, the West demonstrated a sharp increase in liver metastasis mortality from 2014 to 2017 (APC: 11.16%, *P* < 0.001) followed by a nonsignificant trend from 2017 to 2023 (APC: 1.53%, *P* = 0.063). Significant upward trends in lung metastasis mortality were also established in the Midwest from 2011 to 2023 (APC: 4.64%, *P* < 0.001) and in the South from 2013 to 2016 (APC: 10.37%, *P* < 0.001) and from 2016 to 2023 (APC: 2.67%, *P* < 0.001), whereas the West showed a significant spike from 2014 to 2017 (APC: 12.43%, *P* < 0.001) that subsequently lost statistical significance from 2017 to 2023 (APC: 0.40%, *P* = 0.653). CRC mortality is projected to increase from 21.12 in 2024 to 22.56 in 2040 in the South, whereas the rate in the Northeast is projected to decline from 17.00 to 11.99 over the same period. Although liver metastasis mortality in the Northeast initially declined, it increased significantly from 2010 to 2023 (APC: 0.93%, *P* = 0.011) and is projected to continue rising throughout the forecast period, reaching 2.08 by 2040. In the South, liver metastasis-related AAMR is projected to peak earlier and then decline, whereas lung metastasis-related AAMR is expected to rise through 2040. PE-related mortality recorded statistically significant increases in the South, the Midwest, and the West. Sepsis-related mortality demonstrated statistically significant declines in both the Northeast and the Midwest. HF-related AAMR is projected to increase further in the South but remain approximately stable at 0.57 in the Northeast through 2040 (Figure 4B, Table 1, Supplementary Figure S7, and Supplementary Tables S1, S2 and S5).

### Urbanization

Nonmetropolitan areas consistently exhibited higher AAMRs for CRC and most cause-specific categories compared with metropolitan areas. CRC mortality declined in all urbanization categories, but the reduction was less pronounced in nonmetropolitan areas (AAPC, −1.81%) than in large (−2.70%) and medium/small metropolitan areas (−2.33%). Metastatic disease followed a U-shaped trajectory across all regions, with liver and lung metastasis mortality rising after 2010–2013 despite overall negative AAPCs. No significant urban-rural differences were observed for PE-related mortality (all *P* > 0.05). Sepsis-related mortality declined significantly only in large metropolitan areas (AAPC, −1.34%; Table 1 and Supplementary Tables S1, S2).

### Pre-pandemic sensitivity analysis

Restricting the Joinpoint analyses to the pre-pandemic period of 1999 to 2019 materially attenuated several late-period increases observed in the primary analysis (Supplementary Table S3). The increase in pulmonary embolism mortality persisted from 2012 to 2019 (APC: 3.19%, *P* = 0.011), supporting the presence of a pre-pandemic upward trend. Lung metastasis mortality increased significantly from 2014 to 2017 (APC: 9.04%, *P* = 0.004) but was stable from 2017 to 2019 (APC: −0.09%, *P* = 0.976). In contrast, IHD mortality showed no significant increase from 2016 to 2019 (APC: −0.42%, *P* = 0.667), and the positive trend in HF mortality from 2012 to 2019 did not reach statistical significance (APC: 1.35%, *P* = 0.081). Liver metastasis mortality showed positive but nonsignificant changes during 2013 to 2016 (APC: 9.33%, *P* = 0.054) and 2016 to 2019 (APC: 2.74%, *P* = 0.104). No statistically significant pre-pandemic increase in sepsis mortality was identified either in the overall population or among adults aged 65 to 74 years.

## Discussion

This population-based study demonstrates that while overall CRC mortality has declined substantially from 1999 to 2023, the patterns of cause-specific death have undergone a significant shift that reveals critical emerging challenges in cancer survivorship. Metastatic complications, particularly liver and lung involvement, have followed a U-shaped trajectory with mortality rates declining to a nadir around 2013 before rising steadily thereafter, and this burden is increasingly concentrated among younger adults aged 35 to 64 years. Concurrently, HF-related mortality followed a U-shaped trajectory in both sexes, with the reversal beginning in 2014 among women and in 2017 among men; the burden remained particularly high among NH Black individuals and men. Similarly, despite demonstrating a significant downward trajectory when evaluated over the full 25 year duration, ischemic heart disease mortality experienced a critical temporal shift in its later phase, characterized by a statistically significant reversal and upward trend from 2017 to 2023. Pulmonary embolism mortality followed a nonmonotonic trajectory during the early study period but increased significantly and persistently after 2012. Sepsis-related mortality, in contrast, showed an overall modest decline. These findings extend prior work that documented declining overall CRC mortality by illuminating the evolving composition of death causes in an era of prolonged survival.

The pre-pandemic sensitivity analysis refined the interpretation of these late-period changes. PE mortality continued to increase significantly from 2012 to 2019, indicating that this trend preceded the COVID-19 pandemic. Lung metastasis mortality increased significantly from 2014 to 2017 but was stable from 2017 to 2019. In contrast, the late-period increases in IHD, HF, and liver metastasis mortality were attenuated or no longer statistically significant when the analyses were restricted to 1999 to 2019. Similarly, neither the overall population nor adults aged 65 to 74 years exhibited a significant pre-pandemic increase in sepsis mortality. The sharp sepsis increase in the latter group was confined to 2020 to 2023, suggesting that pandemic-era disruptions in cancer diagnosis, treatment continuity, acute care, and cause-of-death certification may have contributed to the observed pattern. Collectively, these findings suggest that some recent trends may reflect both underlying clinical changes and pandemic-related amplification rather than stable long-term trajectories.

The rise in pulmonary embolism aligns with recent national evidence of increasing venous thromboembolism among oncology patients, likely reflecting both greater diagnostic vigilance and the prothrombotic nature of contemporary systemic therapies (50, 51). Notably, unlike prior population-based studies by Dort et al. (29), and Zuin et al. (30) which defined PE as the underlying cause of death to assess PE-related mortality across all cancer types, our analysis designates CRC as the underlying cause of death. This approach provides a more etiologically coherent and clinically relevant framework for understanding how non-cancer complications, including PE, contribute to mortality in the context of prolonged CRC survival. Moreover, our observation of rising metastatic disease, particularly the recent observed increase in lung metastasis mortality among women, whose rate in 2023 exceeded the corresponding 1999 level, is consistent with emerging evidence of an increasing metastatic burden in CRC (3, 52) and may reflect the distinct biology and aggressive clinical course of early-onset CRC, which has surged in recent decades (53, 54).

Several interrelated factors may contribute to these trends. First, therapeutic advances in surgery, chemotherapy, targeted agents, and immune checkpoint inhibitors have extended survival, inadvertently exposing patients to longer durations of risk for metastatic progression and treatment-related cardiotoxicity. Prolonged exposure to treatment increases the cumulative risk of metastatic progression, as residual tumor cells develop acquired resistance under therapeutic pressure, leading to heightened tumor heterogeneity and aggressive biological behavior (55, 56). Potential contributors to the observed cardiovascular trends include longer cancer survival, accumulated cardiometabolic risk, and the cardiotoxic effects of selected systemic therapies. Fluoropyrimidines (57–59), anti-vascular endothelial growth factor agents, multikinase inhibitors, and immune checkpoint inhibitors have each been associated with different forms of cardiovascular toxicity. Oxaliplatin and irinotecan further contribute to systemic toxicity, while monoclonal antibodies such as bevacizumab are associated with hypertension and heart failure through vascular endothelial growth factor inhibition (60–62), and epidermal growth factor receptor inhibitors, like cetuximab and panitumumab, have been linked to arrhythmias, hypomagnesemia, and cardiac dysfunction (63, 64). The multikinase inhibitor regorafenib also elevates the risk of cardiovascular events in solid tumors, including CRC (65, 66). Moreover, immune checkpoint inhibitors, though transformative for select patients, can trigger immune-mediated myocarditis, a rare but potentially fatal irAE mediated by T-cell infiltration of the myocardium (67, 68). However, the pre-pandemic sensitivity analysis showed no significant increase in IHD mortality from 2016 to 2019, indicating that the significant reversal observed from 2017 to 2023 was not evident before the pandemic. Pandemic-related disruptions in cardiovascular and cancer care, competing mortality, and changes in death-certificate attribution may therefore have amplified the observed increase. Because CDC WONDER contains no information on individual treatment exposure, cardiovascular comorbidities, or timing of therapy, treatment-related cardiotoxicity should be regarded as one plausible, nonexclusive hypothesis rather than an established explanation for the observed mortality trend.

Beyond cardiotoxicity, these therapies, combined with the inherent hypercoagulable state of malignancy, heighten the risk of venous thromboembolism (69). Tumor-derived procoagulant factors, therapy-induced endothelial injury, and immune activation synergistically promote thrombogenesis, while imaging surveillance and heightened clinical awareness further improve the detection and death certificate attribution of pulmonary embolism (30). Compounding this risk, a substantial proportion of cancer mortality in the United States is linked to modifiable lifestyle and environmental factors, such as tobacco use, alcohol consumption, obesity, physical inactivity, radiation exposure, occupational carcinogens, and certain infections, many of which independently elevate the likelihood of thrombotic events (70). Second, the increasing burden of CRC among younger adults may contribute to the observed age-specific metastatic patterns. Differences in stage at diagnosis, tumor biology, treatment patterns, and healthcare access are plausible explanations; however, the present dataset contains no tumor genomic, microsatellite, or biomarker information and cannot evaluate these mechanisms directly (71–73). Third, systemic inequities in healthcare access, particularly in the Southern United States and nonmetropolitan areas, result in delayed diagnosis, reduced treatment intensity, and poorer management of comorbid conditions, thereby exacerbating metastatic and heart failure burdens (74, 75).

The pronounced sex-based differences in metastatic patterns, with women showing a recent observed increase in lung metastasis mortality through 2023 and men carrying a higher liver metastasis burden, suggest potential biological differences in CRC dissemination, potentially influenced by hormonal or immune microenvironmental factors. Similarly, the persistent racial disparities, with NH Black individuals bearing the highest HF-related mortality, underscore systemic inequities in cardiovascular risk management among CRC survivors. These findings argue for tailored interventions: enhanced metastatic surveillance for young adults, sex-specific risk stratification, and targeted HF prevention in high-risk racial groups and Southern regions.

## Limitations

This study has several limitations. First, reliance on death certificate data may have resulted in misclassification or underreporting of underlying and contributing conditions, particularly sepsis, HF, PE, and metastatic disease. Secular changes in death-certificate completion, electronic registration, diagnostic detection, and coding practices may also have influenced temporal patterns in recorded contributing conditions. These estimates reflect the recording of selected contributing conditions on death certificates and should not be interpreted as competing-risk estimates or as proof that a contributing condition was the direct cause of death. Sparse counts and CDC WONDER suppression also precluded estimation of PE trends among Hispanic and NH Asian individuals and motivated the exclusion of persons aged 18 to 24 years. In addition, the available 10-year age categories did not permit exact isolation of the conventional early-onset CRC population aged younger than 50 years. Second, the aggregated dataset lacked individual-level information on tumor stage, recurrence, treatment exposure and duration, comorbidities, molecular subtype, microsatellite status, biomarkers, and social determinants. Consequently, adjustment for potential confounders was not possible, and the observed trends cannot establish treatment-specific or tumor-biological mechanisms. Third, ARIMA projections assume continuation of the historical time-series structure and may not capture future changes in screening, treatment, population risk factors, or cause-of-death certification. The widening prediction intervals in several subgroup models further indicate that these projections should be interpreted as conditional scenarios rather than deterministic estimates. Finally, the study period included the COVID-19 pandemic. In the pre-pandemic sensitivity analysis, the increase in PE mortality persisted, whereas late-period increases in IHD, HF, liver metastasis, and sepsis were attenuated or no longer statistically significant. Therefore, some recent trends, particularly the IHD reversal and the sepsis increase among adults aged 65 to 74 years, may have been amplified by pandemic-era effects.

## Conclusions

Although overall CRC mortality declined, the composition of contributing conditions recorded among CRC deaths changed over time. The pre-pandemic persistence of increasing PE mortality warrants further investigation, including prospective studies evaluating risk stratification and evidence-based thromboprophylaxis strategies in appropriately selected patients. Recent increases in IHD, HF, liver metastasis, and sepsis should be interpreted cautiously because they were attenuated after exclusion of pandemic-era observations.

## Data Availability

Publicly available datasets were analyzed in this study. This data can be found at: Repository name: Centers for Disease Control and Prevention Wide-ranging Online Data for Epidemiologic Research (CDC WONDER) Direct, link: https://wonder.cdc.gov/mcd.html, Accession number(s): Not applicable.
