# Supplementary material for: Trends and projections in cause-specific mortality among patients with colorectal cancer in the United States, 1999–2040
Source: Front Public Health. 2026 Aug 25;14:1850707. doi: 10.3389/fpubh.2026.1850707 (PMC13550207; doi:10.3389/fpubh.2026.1850707)
Supplement: Supplementary file 2 [file Table_2.docx]

Supplement 1.

eFigure 1.

eFigure 2.

eFigure 3.

eFigure 4.

eFigure 5.

eFigure 6.

eFigure 7.

eTable 1.

eTable 2.

eTable 3.

eTable 4.

eTable 5.

eTables 4 and 5 are provided in a separate Excel workbook.

**eFigure 1. Geographic Distribution of Colorectal Cancer Deaths, Mortality Rates, and Temporal Trends by State in the United States, 1999-2023.**

**eFigure 2. Observed and Projected Trends in Cause-Specific Age-Adjusted Mortality Rates Among Patients With Colorectal Cancer Stratified by Sex, 1999-2040.**

Solid blue lines represent observed mortality rates from 1999 to 2023; dashed blue lines represent model-based forecasts from 2024 to 2040. Shaded gray areas indicate 95% prediction intervals and are displayed only through the last year in which the lower prediction limit remains nonnegative.

**eFigure 3. Observed and Projected Trends in Cause-Specific Age-Adjusted Mortality Rates Among Patients With Colorectal Cancer Stratified by Race and Ethnicity, 1999-2040.**

Solid blue lines represent observed mortality rates from 1999 to 2023; dashed blue lines represent model-based forecasts from 2024 to 2040. Shaded gray areas indicate 95% prediction intervals and are displayed only through the last year in which the lower prediction limit remains nonnegative.

**eFigure 4. Observed and Projected Trends in Cause-Specific Age-Specific Mortality Rates Among Patients With Colorectal Cancer Stratified by Age (35-44 and 45-54 Years), 1999-2040.**

Solid blue lines represent observed mortality rates from 1999 to 2023; dashed blue lines represent model-based forecasts from 2024 to 2040. Shaded gray areas indicate 95% prediction intervals and are displayed only through the last year in which the lower prediction limit remains nonnegative.

**eFigure 5. Observed and Projected Trends in Cause-Specific Age-Specific Mortality Rates Among Patients With Colorectal Cancer Stratified by Age (55-64 and 65-74 Years), 1999-2040.**

Solid blue lines represent observed mortality rates from 1999 to 2023; dashed blue lines represent model-based forecasts from 2024 to 2040. Shaded gray areas indicate 95% prediction intervals and are displayed only through the last year in which the lower prediction limit remains nonnegative.

**eFigure 6. Observed and Projected Trends in Cause-Specific Age-Specific Mortality Rates Among Patients With Colorectal Cancer Stratified by Age (75-84 and ≥85 Years), 1999-2040.**

Solid blue lines represent observed mortality rates from 1999 to 2023; dashed blue lines represent model-based forecasts from 2024 to 2040. Shaded gray areas indicate 95% prediction intervals and are displayed only through the last year in which the lower prediction limit remains nonnegative.

**eFigure 7. Observed and Projected Trends in Cause-Specific Age-Adjusted Mortality Rates Among Patients With Colorectal Cancer Stratified by Census Region, 1999-2040.**

Solid blue lines represent observed mortality rates from 1999 to 2023; dashed blue lines represent model-based forecasts from 2024 to 2040. Shaded gray areas indicate 95% prediction intervals and are displayed only through the last year in which the lower prediction limit remains nonnegative.

| **eTable 1. Observed Trends in Nonmetastatic Disease-Specific Mortality With Colorectal Cancer, 1999-2023** | | | | | | | | | | | | |
| --- | --- | --- | --- | --- | --- | --- | --- | --- | --- | --- | --- | --- |
| **Characteristic** | **Heart Failure** | | | **Ischemic Heart Disease** | | | **Pulmonary Embolism** | | | **Sepsis** | | |
|  | **AAMR (95% CI)** | | **AAPC,**  **P value** | **AAMR (95% CI)** | | **AAPC,**  **P value** | **AAMR (95% CI)** | | **AAPC,**  **P value** | **AAMR (95% CI)** | | **AAPC,**  **P value** |
|  | **1999** | **2023** |  | **1999** | **2023** |  | **1999** | **2023** |  | **1999** | **2023** |  |
| **Sex** | | | | | | | | | | | | |
| Both | 1.00 (0.95-1.05) | 0.67 (0.64-0.70) | -1.42 (-1.78 to -1.02), P<.001 | 1.72 (1.66-1.78) | 0.80 (0.77-0.84) | -3.02 (-3.19 to -2.85), P<.001 | 0.29 (0.26-0.31) | 0.38 (0.36-0.40) | 1.08 (0.80 to 1.38), P<.001 | 1.15 (1.10-1.20) | 1.10 (1.06-1.14) | -0.37 (-0.67 to -0.07), P=0.014 |
| Female | 0.85 (0.79-0.90) | 0.49 (0.46-0.53) | -2.03 (-2.38 to -1.71), P<.001 | 1.20 (1.14-1.27) | 0.50 (0.47-0.54) | -3.60 (-3.93 to -3.28), P<.001 | 0.27  (0.24-0.30) | 0.36 (0.32-0.39) | 1.34 (0.74 to 1.97), P<.001 | 0.96  (0.90-1.01) | 0.90  (0.85-0.95) | -0.40 (-0.68 to -0.10), P=0.010 |
| Male | 1.15 (1.07-1.24) | 0.91 (0.85-0.96) | -0.81 (-1.14 to -0.36), P<.001 | 2.49 (2.37-2.61) | 1.19 (1.12-1.25) | -2.81 (-3.15 to -2.36), P<.001 | 0.32  (0.28-0.37) | 0.44 (0.40-0.48) | 0.73 (0.26 to 1.33), P=0.003 | 1.47  (1.38-1.56) | 1.32  (1.26-1.39) | -0.50 (-0.75 to -0.25), P<.001 |
| **Race** | | | | | | | | | |  | | |
| Hispanic | 0.46 (0.31-0.65) | 0.35 (0.28-0.43) | -1.47 (-2.40 to -0.59), P<.001 | 1.18 (0.94-1.46) | 0.55 (0.46-0.64) | -2.94 (-3.60 to -2.15), P<.001 | NA | NA | NA | 1.04  (0.83-1.28) | 1.06  (0.94-1.17) | 0.12 (-0.32 to 0.71), P=0.475 |
| NH Asian | NA | 0.33 (0.24-0.43) | -2.29 (-3.63 to -0.80), P=0.003 | 0.61 (0.37-0.94) | 0.35 (0.26-0.46) | -3.45 (-4.49 to -2.29), P<.001 | NA | NA | NA | 0.91 (0.63-1.26) | 0.93  (0.78-1.08) | 0.11 (-0.41 to 0.80), P=0.545 |
| NH Black | 1.28 (1.09-1.47) | 0.99 (0.86-1.11) | -1.15 (-1.56 to -0.57), P<.001 | 1.75 (1.53-1.97) | 0.90 (0.78-1.01) | -3.25 (-4.32 to -2.62), P<.001 | 0.58 (0.46-0.72) | 0.69 (0.59-0.79) | 0.51 (-0.10 to 1.21), P=0.097 | 1.86 (1.64-2.08) | 1.66 (1.51-1.82) | -0.63 (-1.30 to -0.14), P=0.014 |
| NH White | 0.98 (0.93-1.03) | 0.71 (0.67-0.74) | -1.18 (-1.62 to -0.73), P<.001 | 1.77 (1.70-1.84) | 0.85 (0.81-0.89) | -2.90 (-3.09 to -2.69), P<.001 | 0.27 (0.24-0.29) | 0.40 (0.37-0.43) | 1.47 (0.94 to 1.99), P<.001 | 1.11 (1.06-1.17) | 1.00 (0.96-1.05) | -0.49 (-0.85 to -0.13), P=0.008 |
| **Age Group** | | | | | | | | | |  | | |
| 35-44 years | NA | NA | NA | NA | NA | NA | NA | NA | NA | 0.08 (0.06-0.12) | 0.26 (0.22-0.31) | 4.11 (3.27 to 5.18), P<.001 |
| 45-54 years | NA | NA | NA | 0.09 (0.06-0.13) | 0.12 (0.09-0.15) | 1.77 (0.44 to 3.28), P=0.014 | 0.11 (0.08-0.16) | 0.25 (0.20-0.30) | 4.24 (3.51 to 5.12), P<.001 | 0.35 (0.29-0.41) | 0.78 (0.70-0.87) | 3.65 (3.08 to 4.42), P<.001 |
| 55-64 years | 0.37 (0.29-0.45) | 0.42 (0.36-0.49) | 1.62 (0.99 to 2.32), P<.001 | 0.56 (0.46-0.65) | 0.56 (0.49-0.64) | -0.74 (-1.32 to -0.19), P=0.009 | 0.34 (0.27-0.43) | 0.52 (0.45-0.59) | 1.46 (0.54 to 2.45), P=0.002 | 1.28 (1.13-1.42) | 1.62 (1.50-1.74) | 1.16 (0.64 to 1.78), P<.001 |
| 65-74 years | 1.25 (1.09-1.42) | 1.18 (1.07-1.30) | -0.45 (-0.92 to 0.05), P=0.078 | 3.35 (3.09-3.61) | 1.59 (1.46-1.72) | -3.16 (-3.62 to -2.71), P<.001 | 0.79 (0.66-0.92) | 0.85 (0.75-0.95) | 0.37 (-0.23 to 1.35), P=0.154 | 2.86 (2.61-3.10) | 2.59 (2.42-2.76) | -0.45 (-0.78 to -0.12), P=0.022 |
| 75-84 years | 4.97 (4.58-5.37) | 3.32 (3.06-3.58) | -1.64 (-2.12 to -1.19), P<.001 | 10.09 (9.53-10.66) | 3.85 (3.57-4.14) | -3.55 (-3.84 to -3.25), P<.001 | 1.46 (1.24-1.67) | 1.45 (1.27-1.62) | -0.13 (-0.96 to 0.48), P=0.763 | 5.45 (5.03-5.86) | 3.63 (3.36-3.91) | -1.94 (-2.29 to -1.60), P<.001 |
| 85+ years | 18.46 (17.16-19.77) | 10.09 (9.30-10.88) | -2.57 (-2.97 to -2.18), P<.001 | 24.27 (22.77-25.76) | 11.12 (10.29-11.95) | -3.21 (-3.44 to -2.98), P<.001 | 1.56 (1.21-1.99) | 2.36 (1.97-2.74) | 1.25 (0.41 to 2.78), P=0.025 | 9.05 (8.14-9.97) | 5.13 (4.57-5.70) | -2.50 (-3.02 to -2.03), P<.001 |
| **Census Region** | | | | | | | | | |  | | |
| Midwest | 1.10 (1.00-1.20) | 0.77 (0.70-0.84) | -1.63 (-2.36 to -0.94), P<.001 | 1.95 (1.82-2.08) | 0.88 (0.80-0.95) | -3.20 (-3.53 to -2.83), P<.001 | 0.32 (0.27-0.37) | 0.40 (0.34-0.45) | 1.17 (0.28 to 1.92), P=0.022 | 1.01 (0.91-1.10) | 0.94 (0.86-1.02) | -0.42 (-0.81 to -0.01), P=0.044 |
| Northeast | 1.02 (0.92-1.12) | 0.57 (0.50-0.63) | -2.65 (-3.26 to -2.10), P<.001 | 2.23 (2.08-2.38) | 0.58 (0.51-0.64) | -5.17 (-5.88 to -4.66), P<.001 | 0.34 (0.28-0.40) | 0.36 (0.30-0.42) | 0.33 (-0.47 to 1.21), P=0.394 | 1.37 (1.25-1.49) | 0.95 (0.86-1.04) | -1.46 (-1.86 to -1.09), P<.001 |
| South | 0.92 (0.85-1.00) | 0.74 (0.68-0.79) | -0.90 (-1.21 to -0.59), P<.001 | 1.44 (1.34-1.53) | 0.95 (0.89-1.01) | -1.55 (-1.87 to -1.12), P<.001 | 0.26 (0.22-0.30) | 0.42 (0.38-0.46) | 1.29 (0.53 to 2.08), P=0.001 | 1.14 (1.05-1.22) | 1.17 (1.11-1.24) | 0.12 (-0.02 to 0.26), P=0.088 |
| West | 0.86 (0.77-0.96) | 0.59 (0.53-0.65) | -1.23 (-1.74 to -0.72), P<.001 | 1.39 (1.26-1.51) | 0.67 (0.60-0.73) | -2.86 (-3.33 to -2.47), P<.001 | 0.25 (0.20-0.31) | 0.39 (0.34-0.44) | 1.64 (0.79 to 2.27), P<.001 | 1.13 (1.02-1.25) | 1.19 (1.10-1.28) | 0.53 (-0.09 to 0.98), P=0.085 |
| **Urbanization^1^** | | | | | | | | | |  | | |
| Large Metro | 0.92 (0.86-0.99) | 0.53 (0.49-0.57) | -2.62 (-2.99 to -2.17), P<.001 | 1.71 (1.62-1.79) | 0.64 (0.60-0.69) | -4.53 (-4.87 to -4.25), P<.001 | 0.29 (0.26-0.33) | 0.32 (0.29-0.35) | 0.43 (-0.29 to 1.09), P=0.230 | 1.30 (1.22-1.37) | 0.95 (0.89-1.00) | -1.34 (-1.66 to -0.91), P<.001 |
| Medium-Small Metro | 0.90 (0.82-0.98) | 0.65 (0.60-0.71) | -1.81 (-2.35 to -1.33), P<.001 | 1.62 (1.51-1.73) | 0.87 (0.81-0.93) | -3.23 (-3.85 to -2.70), P<.001 | 0.26 (0.22-0.31) | 0.29 (0.25-0.32) | 0.71 (-0.04 to 1.59), P=0.066 | 0.97 (0.89-1.06) | 0.86 (0.79-0.92) | -0.46 (-0.87 to 0.02), P=0.057 |
| Nonmetropolitan | 1.24 (1.12-1.36) | 0.94 (0.85-1.03) | -2.12 (-3.00 to -1.37), P<.001 | 1.92 (1.77-2.06) | 1.09 (0.99-1.19) | -2.75 (-3.06 to -2.38), P<.001 | 0.33 (0.27-0.40) | 0.37 (0.31-0.43) | 0.30 (-0.52 to 1.24), P=0.460 | 1.09 (0.98-1.21) | 1.06 (0.96-1.16) | 0.21 (-0.29 to 0.76), P=0.378 |
| ^1^ Urbanization strata were available through 2020 only; thus 2020 AAMRs are shown in the 2023 column for these rows. | | | | | | | | | | | | |
| Abbreviations: AAMR, age-adjusted mortality rate; AAPC, average annual percent change; CI, confidence interval; NH, non-Hispanic. | | | | | | | | | | | | |
| NA indicates that the mortality rate or AAPC could not be estimated because one or more required annual cells contained 0 to 9 deaths and were suppressed by CDC WONDER. | | | | | | | | | | | | |

**eTable 2. Annual Percent Changes in Cause-Specific Age-Adjusted Mortality Rates Among Patients With Colorectal Cancer**

| **Characteristic** | **Temporal trend difference** | | **P value** |
| --- | --- | --- | --- |
|  | **Temporal segment** | **APC (95% CI), %** |  |
| **Sex** | | | |
| **Both** | | | |
| Colorectal Cancer | 1999-2012 | -2.77 (-3.42 to -2.51) | P<.001 |
|  | 2012-2020 | -1.79 (-2.90 to -1.24) | P<.001 |
|  | 2020-2023 | 0.13 (-1.36 to 1.78) | P=0.850 |
| Heart Failure | 1999-2005 | -3.15 (-4.74 to 1.87) | P=0.098 |
|  | 2005-2013 | -5.63 (-10.30 to 5.24) | P=0.179 |
|  | 2013-2023 | 3.16 (1.36 to 5.28) | P=0.033 |
| Ischemic Heart Disease | 1999-2005 | -3.06 (-3.80 to -1.85) | P=0.005 |
|  | 2005-2017 | -5.25 (-5.78 to -4.94) | P<.001 |
|  | 2017-2023 | 1.64 (0.68 to 3.01) | P=0.001 |
| Liver Metastasis | 1999-2008 | -7.89 (-8.61 to -7.38) | P<.001 |
|  | 2008-2013 | -0.21 (-3.28 to 2.05) | P=0.663 |
|  | 2013-2016 | 9.31 (6.35 to 11.09) | P<.001 |
|  | 2016-2023 | 2.89 (1.98 to 3.43) | P=0.002 |
| Lung Metastasis | 1999-2008 | -3.80 (-4.78 to -3.20) | P=0.002 |
|  | 2008-2014 | 1.40 (-1.18 to 2.90) | P=0.154 |
|  | 2014-2017 | 8.33 (5.47 to 9.88) | P<.001 |
|  | 2017-2023 | 2.08 (0.77 to 2.78) | P=0.013 |
| Pulmonary Embolism | 1999-2004 | -1.95 (-6.36 to 0.10) | P=0.059 |
|  | 2004-2008 | 4.26 (1.50 to 7.83) | P=0.032 |
|  | 2008-2012 | -4.86 (-8.31 to -1.88) | P=0.032 |
|  | 2012-2023 | 3.59 (2.99 to 4.37) | P=0.003 |
| Sepsis | 1999-2013 | -1.58 (-2.50 to -1.01) | P<.001 |
|  | 2013-2023 | 1.33 (0.47 to 2.89) | P=0.004 |
| **Female** | | | |
| Colorectal Cancer | 1999-2012 | -2.80 (-3.82 to -0.82) | P=0.024 |
|  | 2012-2020 | -1.90 (-3.98 to -1.12) | P=0.004 |
|  | 2020-2023 | 0.32 (-1.60 to 2.37) | P=0.755 |
| Heart Failure | 1999-2014 | -4.96 (-5.66 to -4.42) | P<.001 |
|  | 2014-2023 | 3.05 (1.72 to 4.80) | P<.001 |
| Ischemic Heart Disease | 1999-2006 | -3.55 (-4.61 to -1.24) | P=0.022 |
|  | 2006-2017 | -6.52 (-8.38 to -5.83) | P<.001 |
|  | 2017-2023 | 1.94 (-0.03 to 4.78) | P=0.055 |
| Liver Metastasis | 1999-2008 | -7.65 (-8.56 to -7.09) | P<.001 |
|  | 2008-2013 | -1.31 (-4.36 to 1.33) | P=0.193 |
|  | 2013-2016 | 10.39 (6.93 to 12.43) | P<.001 |
|  | 2016-2023 | 2.74 (1.70 to 3.37) | P=0.004 |
| Lung Metastasis | 1999-2006 | -4.00 (-7.54 to -2.72) | P=0.002 |
|  | 2006-2014 | -0.25 (-1.64 to 2.35) | P=0.691 |
|  | 2014-2017 | 11.36 (6.82 to 13.65) | P=0.007 |
|  | 2017-2023 | 1.77 (-0.25 to 2.89) | P=0.070 |
| Pulmonary Embolism | 1999-2015 | -0.81 (-2.38 to 0.18) | P=0.106 |
|  | 2015-2023 | 5.79 (3.41 to 11.94) | P<.001 |
| Sepsis | 1999-2013 | -1.75 (-2.55 to -1.19) | P<.001 |
|  | 2013-2023 | 1.54 (0.65 to 3.00) | P<.001 |
| **Male** | | | |
| Colorectal Cancer | 1999-2002 | -2.00 (-2.59 to -0.72) | P=0.013 |
|  | 2002-2005 | -4.01 (-4.47 to -3.25) | P<.001 |
|  | 2005-2013 | -2.52 (-2.96 to -1.82) | P<.001 |
|  | 2013-2019 | -1.92 (-2.57 to -1.04) | P=0.002 |
|  | 2019-2023 | -0.44 (-1.10 to 0.78) | P=0.319 |
| Heart Failure | 1999-2006 | -2.30 (-4.31 to 2.55) | P=0.124 |
|  | 2006-2011 | -5.83 (-9.68 to -0.51) | P=0.040 |
|  | 2011-2017 | -0.78 (-4.13 to 4.70) | P=0.734 |
|  | 2017-2023 | 5.40 (3.18 to 10.11) | P=0.016 |
| Ischemic Heart Disease | 1999-2006 | -3.02 (-4.24 to 1.19) | P=0.066 |
|  | 2006-2016 | -5.10 (-10.01 to -2.20) | P=0.032 |
|  | 2016-2023 | 0.78 (-1.15 to 4.84) | P=0.245 |
| Liver Metastasis | 1999-2007 | -8.49 (-9.27 to -7.92) | P<.001 |
|  | 2007-2013 | -1.02 (-2.66 to 0.46) | P=0.116 |
|  | 2013-2016 | 9.02 (6.22 to 10.62) | P<.001 |
|  | 2016-2023 | 2.91 (2.10 to 3.47) | P<.001 |
| Lung Metastasis | 1999-2008 | -4.36 (-4.95 to -3.94) | P<.001 |
|  | 2008-2013 | 0.86 (-1.03 to 2.35) | P=0.266 |
|  | 2013-2017 | 6.21 (4.82 to 8.02) | P<.001 |
|  | 2017-2023 | 1.97 (1.20 to 2.56) | P=0.001 |
| Pulmonary Embolism | 1999-2003 | -4.44 (-12.93 to 0.83) | P=0.080 |
|  | 2003-2007 | 6.20 (-2.29 to 12.51) | P=0.060 |
|  | 2007-2013 | -3.60 (-9.55 to 1.89) | P=0.062 |
|  | 2013-2023 | 3.42 (2.17 to 5.35) | P=0.015 |
| Sepsis | 1999-2012 | -1.66 (-2.51 to -1.12) | P<.001 |
|  | 2012-2023 | 0.88 (0.25 to 1.87) | P=0.005 |
| **Race** | | | |
| **Hispanic** | | | |
| Colorectal Cancer | 1999-2019 | -1.68 (-1.96 to -1.52) | P=0.001 |
|  | 2019-2023 | 0.43 (-0.90 to 2.81) | P=0.456 |
| Heart Failure | 1999-2016 | -3.39 (-6.60 to -2.20) | P<.001 |
|  | 2016-2023 | 3.36 (-0.20 to 14.41) | P=0.066 |
| Ischemic Heart Disease | 1999-2023 | -2.94 (-3.60 to -2.15) | P<.001 |
| Liver Metastasis | 1999-2008 | -5.59 (-10.21 to 0.84) | P=0.057 |
|  | 2008-2020 | 4.06 (-9.61 to 11.20) | P=0.165 |
|  | 2020-2023 | -1.34 (-8.46 to 3.52) | P=0.719 |
| Lung Metastasis | 1999-2004 | -7.16 (-18.82 to -0.43) | P=0.040 |
|  | 2004-2013 | 1.83 (-2.92 to 12.12) | P=0.270 |
|  | 2013-2016 | 14.01 (-4.22 to 18.63) | P=0.104 |
|  | 2016-2023 | -0.08 (-3.60 to 5.12) | P=0.843 |
| Sepsis | 1999-2023 | 0.12 (-0.32 to 0.71) | P=0.475 |
| **NH American Indian** | | | |
| Colorectal Cancer | 1999-2023 | -0.92 (-1.36 to -0.40) | P<.001 |
| Liver Metastasis | 2010-2023 | 2.06 (-0.62 to 5.15) | P=0.118 |
| **NH Asian** | | | |
| Colorectal Cancer | 1999-2023 | -1.68 (-1.95 to -1.35) | P<.001 |
| Heart Failure | 2003-2016 | -5.96 (-10.54 to -3.75) | P<.001 |
|  | 2016-2023 | 4.90 (0.40 to 19.91) | P=0.038 |
| Ischemic Heart Disease | 1999-2023 | -3.45 (-4.49 to -2.29) | P<.001 |
| Liver Metastasis | 1999-2006 | -9.58 (-16.19 to -5.66) | P<.001 |
|  | 2006-2023 | 3.21 (2.37 to 4.50) | P<.001 |
| Lung Metastasis | 1999-2004 | -6.44 (-23.25 to 0.95) | P=0.103 |
|  | 2004-2023 | 2.88 (2.22 to 5.01) | P=0.005 |
| Sepsis | 1999-2023 | 0.11 (-0.41 to 0.80) | P=0.545 |
| **NH Black** | | | |
| Colorectal Cancer | 1999-2019 | -2.72 (-3.46 to -2.55) | P=0.014 |
|  | 2019-2023 | -1.28 (-2.59 to 1.11) | P=0.222 |
| Heart Failure | 1999-2001 | -16.95 (-22.57 to -9.02) | P<.001 |
|  | 2001-2004 | 4.99 (-1.17 to 9.43) | P=0.097 |
|  | 2004-2016 | -4.09 (-5.78 to -3.32) | P=0.015 |
|  | 2016-2023 | 6.64 (4.78 to 9.04) | P=0.002 |
| Ischemic Heart Disease | 1999-2019 | -5.17 (-6.73 to -4.46) | P=0.004 |
|  | 2019-2023 | 6.96 (-2.03 to 22.64) | P=0.122 |
| Liver Metastasis | 1999-2009 | -7.86 (-9.41 to -6.65) | P<.001 |
|  | 2009-2023 | 2.55 (1.77 to 3.54) | P<.001 |
| Lung Metastasis | 1999-2009 | -3.34 (-8.53 to -1.45) | P<.001 |
|  | 2009-2023 | 2.23 (1.24 to 4.29) | P<.001 |
| Pulmonary Embolism | 1999-2023 | 0.51 (-0.10 to 1.21) | P=0.097 |
| Sepsis | 1999-2019 | -1.62 (-3.14 to -1.10) | P=0.011 |
|  | 2019-2023 | 4.47 (-0.31 to 13.37) | P=0.077 |
| **NH White** | | | |
| Colorectal Cancer | 1999-2011 | -2.84 (-3.17 to -2.66) | P<.001 |
|  | 2011-2020 | -1.66 (-2.23 to -1.23) | P<.001 |
|  | 2020-2023 | 0.86 (-0.57 to 2.78) | P=0.213 |
| Heart Failure | 1999-2004 | -2.53 (-4.74 to 3.94) | P=0.143 |
|  | 2004-2013 | -5.64 (-10.73 to 5.42) | P=0.147 |
|  | 2013-2023 | 3.72 (2.08 to 5.64) | P=0.035 |
| Ischemic Heart Disease | 1999-2006 | -3.13 (-3.86 to -1.97) | P=0.007 |
|  | 2006-2016 | -5.45 (-6.57 to -4.97) | P=0.002 |
|  | 2016-2023 | 1.12 (0.16 to 2.40) | P=0.032 |
| Liver Metastasis | 1999-2007 | -8.23 (-8.79 to -7.84) | P<.001 |
|  | 2007-2013 | -1.59 (-2.46 to -0.46) | P=0.012 |
|  | 2013-2016 | 10.20 (7.98 to 11.50) | P<.001 |
|  | 2016-2023 | 3.67 (3.06 to 4.12) | P<.001 |
| Lung Metastasis | 1999-2009 | -3.61 (-4.60 to -3.09) | P=0.004 |
|  | 2009-2014 | 1.91 (-2.09 to 3.82) | P=0.228 |
|  | 2014-2017 | 8.55 (5.55 to 10.24) | P=0.002 |
|  | 2017-2023 | 2.84 (1.40 to 3.62) | P=0.010 |
| Pulmonary Embolism | 1999-2013 | 0.00 (-2.19 to 0.93) | P=0.893 |
|  | 2013-2023 | 3.56 (2.18 to 7.21) | P<.001 |
| Sepsis | 1999-2012 | -1.86 (-3.37 to -1.12) | P<.001 |
|  | 2012-2023 | 1.16 (0.26 to 3.20) | P=0.012 |
| **Age Group** | | | |
| **25-34 years** | | | |
| Colorectal Cancer | 1999-2023 | 0.45 (-0.01 to 0.95) | P=0.052 |
| **35-44 years** | | | |
| Colorectal Cancer | 1999-2023 | 1.03 (0.85 to 1.21) | P<.001 |
| Liver Metastasis | 1999-2009 | -3.12 (-5.83 to -1.23) | P=0.001 |
|  | 2009-2023 | 8.08 (7.03 to 9.51) | P<.001 |
| Lung Metastasis | 1999-2007 | 0.11 (-10.43 to 4.11) | P=0.958 |
|  | 2007-2023 | 7.89 (6.89 to 10.40) | P<.001 |
| Sepsis | 1999-2006 | -0.83 (-13.24 to 3.63) | P=0.655 |
|  | 2006-2023 | 6.21 (5.32 to 9.01) | P=0.002 |
| **45-54 years** | | | |
| Colorectal Cancer | 1999-2001 | 2.82 (-1.49 to 6.04) | P=0.113 |
|  | 2001-2004 | -4.02 (-4.94 to 1.08) | P=0.093 |
|  | 2004-2023 | 0.92 (0.74 to 1.12) | P=0.018 |
| Ischemic Heart Disease | 1999-2023 | 1.77 (0.44 to 3.28) | P=0.014 |
| Liver Metastasis | 1999-2006 | -6.63 (-12.32 to -4.45) | P=0.013 |
|  | 2006-2013 | 0.80 (-3.32 to 5.42) | P=0.644 |
|  | 2013-2016 | 16.26 (8.77 to 20.46) | P=0.012 |
|  | 2016-2023 | 5.39 (1.90 to 7.14) | P=0.021 |
| Lung Metastasis | 1999-2005 | -4.23 (-12.63 to -0.56) | P=0.032 |
|  | 2005-2013 | 3.44 (-0.52 to 9.15) | P=0.071 |
|  | 2013-2017 | 14.69 (8.90 to 20.62) | P=0.013 |
|  | 2017-2023 | 5.25 (1.56 to 7.37) | P=0.023 |
| Pulmonary Embolism | 1999-2023 | 4.24 (3.51 to 5.12) | P<.001 |
| Sepsis | 1999-2009 | 1.96 (-5.24 to 3.93) | P=0.312 |
|  | 2009-2023 | 4.88 (3.96 to 9.32) | P=0.022 |
| **55-64 years** | | | |
| Colorectal Cancer | 1999-2005 | -3.60 (-5.78 to -2.64) | P=0.007 |
|  | 2005-2013 | -1.37 (-2.70 to -0.51) | P=0.016 |
|  | 2013-2023 | -0.15 (-0.59 to 1.26) | P=0.651 |
| Heart Failure | 1999-2014 | -1.69 (-3.35 to -0.43) | P=0.010 |
|  | 2014-2023 | 7.38 (5.23 to 10.65) | P<.001 |
| Ischemic Heart Disease | 1999-2017 | -2.75 (-3.72 to -2.01) | P<.001 |
|  | 2017-2023 | 5.55 (2.24 to 12.20) | P=0.002 |
| Liver Metastasis | 1999-2006 | -8.30 (-11.45 to -6.83) | P<.001 |
|  | 2006-2013 | -1.63 (-4.19 to 1.28) | P=0.160 |
|  | 2013-2016 | 13.80 (8.75 to 16.73) | P<.001 |
|  | 2016-2023 | 3.42 (1.87 to 4.37) | P=0.006 |
| Lung Metastasis | 1999-2006 | -3.66 (-10.34 to -1.03) | P=0.036 |
|  | 2006-2013 | 0.61 (-2.09 to 14.98) | P=0.536 |
|  | 2013-2016 | 14.41 (-0.16 to 17.82) | P=0.055 |
|  | 2016-2023 | 3.07 (0.19 to 6.95) | P=0.046 |
| Pulmonary Embolism | 1999-2010 | -1.05 (-10.07 to 1.22) | P=0.359 |
|  | 2010-2023 | 3.64 (2.29 to 9.34) | P=0.006 |
| Sepsis | 1999-2010 | -0.88 (-5.42 to 0.66) | P=0.235 |
|  | 2010-2023 | 2.93 (2.04 to 5.21) | P<.001 |
| **65-74 years** | | | |
| Colorectal Cancer | 1999-2001 | -0.96 (-2.56 to 0.58) | P=0.124 |
|  | 2001-2004 | -4.98 (-5.59 to -4.00) | P<.001 |
|  | 2004-2014 | -3.52 (-3.82 to -2.76) | P<.001 |
|  | 2014-2020 | -2.04 (-3.30 to -1.35) | P<.001 |
|  | 2020-2023 | 0.05 (-1.20 to 1.56) | P=0.894 |
| Heart Failure | 1999-2015 | -3.21 (-4.22 to -2.36) | P<.001 |
|  | 2015-2023 | 5.29 (3.31 to 8.32) | P<.001 |
| Ischemic Heart Disease | 1999-2018 | -4.70 (-5.34 to -4.26) | P<.001 |
|  | 2018-2023 | 2.94 (-0.40 to 10.81) | P=0.078 |
| Liver Metastasis | 1999-2008 | -9.33 (-10.89 to -8.50) | P<.001 |
|  | 2008-2014 | -0.68 (-6.04 to 1.79) | P=0.374 |
|  | 2014-2017 | 9.22 (4.60 to 12.01) | P=0.011 |
|  | 2017-2023 | 1.40 (-0.73 to 2.44) | P=0.134 |
| Lung Metastasis | 1999-2013 | -3.42 (-4.49 to -2.57) | P=0.021 |
|  | 2013-2016 | 9.40 (-3.85 to 12.52) | P=0.090 |
|  | 2016-2023 | 1.38 (-1.45 to 3.05) | P=0.149 |
| Pulmonary Embolism | 1999-2008 | 0.65 (-1.64 to 11.74) | P=0.418 |
|  | 2008-2013 | -6.15 (-13.66 to 3.73) | P=0.074 |
|  | 2013-2023 | 3.54 (1.13 to 7.43) | P=0.040 |
| Sepsis | 1999-2012 | -2.17 (-3.41 to -1.02) | P=0.039 |
|  | 2012-2017 | 1.97 (-3.05 to 5.65) | P=0.107 |
|  | 2017-2020 | -4.10 (-6.73 to -0.09) | P=0.039 |
|  | 2020-2023 | 7.08 (3.68 to 13.16) | P=0.001 |
| **75-84 years** | | | |
| Colorectal Cancer | 1999-2023 | -3.20 (-3.29 to -3.12) | P<.001 |
| Heart Failure | 1999-2015 | -4.18 (-5.16 to -3.46) | P<.001 |
|  | 2015-2023 | 3.62 (1.49 to 7.95) | P=0.004 |
| Ischemic Heart Disease | 1999-2006 | -2.95 (-3.95 to -0.91) | P=0.027 |
|  | 2006-2016 | -5.69 (-9.09 to -4.98) | P<.001 |
|  | 2016-2023 | -1.03 (-2.47 to 1.63) | P=0.265 |
| Liver Metastasis | 1999-2009 | -8.10 (-9.23 to -7.28) | P<.001 |
|  | 2009-2023 | 1.97 (1.37 to 2.71) | P<.001 |
| Lung Metastasis | 1999-2008 | -4.35 (-6.29 to -3.19) | P<.001 |
|  | 2008-2023 | 0.85 (0.26 to 1.63) | P=0.007 |
| Pulmonary Embolism | 1999-2018 | -1.28 (-3.94 to -0.59) | P=0.011 |
|  | 2018-2023 | 4.35 (0.02 to 15.42) | P=0.048 |
| Sepsis | 1999-2023 | -1.94 (-2.29 to -1.60) | P<.001 |
| **85+ years** | | | |
| Colorectal Cancer | 1999-2018 | -2.96 (-3.16 to -2.79) | P<.001 |
|  | 2018-2023 | -0.02 (-1.23 to 2.20) | P=0.997 |
| Heart Failure | 1999-2013 | -5.51 (-6.45 to -4.77) | P<.001 |
|  | 2013-2023 | 1.70 (0.33 to 3.46) | P=0.016 |
| Ischemic Heart Disease | 1999-2007 | -4.17 (-4.82 to -2.78) | P=0.005 |
|  | 2007-2014 | -6.22 (-8.73 to -5.27) | P<.001 |
|  | 2014-2019 | -2.61 (-5.16 to -0.10) | P=0.046 |
|  | 2019-2023 | 3.56 (1.43 to 7.81) | P=0.003 |
| Liver Metastasis | 1999-2010 | -6.38 (-7.45 to -5.53) | P<.001 |
|  | 2010-2023 | 3.27 (2.57 to 4.09) | P<.001 |
| Lung Metastasis | 1999-2010 | -3.72 (-6.68 to -2.22) | P<.001 |
|  | 2010-2023 | 2.89 (1.79 to 4.72) | P<.001 |
| Pulmonary Embolism | 1999-2008 | 2.75 (-0.26 to 18.84) | P=0.057 |
|  | 2008-2013 | -6.56 (-16.03 to -0.35) | P=0.045 |
|  | 2013-2023 | 4.00 (1.03 to 11.90) | P=0.034 |
| Sepsis | 1999-2016 | -3.44 (-4.91 to -2.96) | P<.001 |
|  | 2016-2023 | -0.16 (-2.18 to 5.90) | P=0.959 |
| **Census Region** | | | |
| **Midwest** | | | |
| Colorectal Cancer | 1999-2012 | -2.85 (-3.16 to -2.64) | P<.001 |
|  | 2012-2023 | -1.48 (-1.78 to -1.05) | P<.001 |
| Heart Failure | 1999-2014 | -4.64 (-6.54 to -3.45) | P<.001 |
|  | 2014-2023 | 3.60 (0.99 to 10.04) | P=0.010 |
| Ischemic Heart Disease | 1999-2007 | -2.99 (-3.97 to -1.05) | P=0.026 |
|  | 2007-2015 | -6.55 (-11.43 to -5.34) | P=0.004 |
|  | 2015-2023 | 0.07 (-1.39 to 2.40) | P=0.883 |
| Liver Metastasis | 1999-2009 | -8.49 (-11.16 to -6.72) | P=0.003 |
|  | 2009-2013 | -1.40 (-11.77 to 7.79) | P=0.306 |
|  | 2013-2016 | 11.25 (-2.01 to 15.03) | P=0.080 |
|  | 2016-2023 | 4.62 (1.74 to 6.80) | P=0.030 |
| Lung Metastasis | 1999-2011 | -3.62 (-4.81 to -2.67) | P<.001 |
|  | 2011-2023 | 4.64 (3.75 to 5.81) | P<.001 |
| Pulmonary Embolism | 1999-2017 | -0.23 (-3.25 to 0.61) | P=0.511 |
|  | 2017-2023 | 5.48 (1.65 to 17.49) | P=0.002 |
| Sepsis | 1999-2023 | -0.42 (-0.81 to -0.01) | P=0.044 |
| **Northeast** | | | |
| Colorectal Cancer | 1999-2009 | -3.75 (-4.18 to -3.48) | P<.001 |
|  | 2009-2023 | -2.60 (-2.79 to -2.34) | P<.001 |
| Heart Failure | 1999-2014 | -5.06 (-6.44 to -4.18) | P<.001 |
|  | 2014-2023 | 1.50 (-0.74 to 5.61) | P=0.188 |
| Ischemic Heart Disease | 1999-2007 | -4.46 (-5.80 to -1.18) | P=0.039 |
|  | 2007-2019 | -7.29 (-13.88 to -6.40) | P=0.006 |
|  | 2019-2023 | -0.01 (-5.61 to 10.40) | P=0.987 |
| Liver Metastasis | 1999-2010 | -9.21 (-10.06 to -8.55) | P<.001 |
|  | 2010-2023 | 0.93 (0.23 to 1.75) | P=0.011 |
| Lung Metastasis | 1999-2010 | -4.94 (-6.05 to -4.13) | P<.001 |
|  | 2010-2023 | 0.14 (-0.56 to 1.08) | P=0.646 |
| Pulmonary Embolism | 1999-2023 | 0.33 (-0.47 to 1.21) | P=0.394 |
| Sepsis | 1999-2019 | -2.68 (-3.10 to -2.35) | P<.001 |
|  | 2019-2023 | 4.83 (1.16 to 12.52) | P=0.009 |
| **South** | | | |
| Colorectal Cancer | 1999-2012 | -2.43 (-3.25 to -2.18) | P=0.008 |
|  | 2012-2020 | -1.57 (-2.35 to -0.82) | P=0.002 |
|  | 2020-2023 | 0.90 (-0.74 to 2.86) | P=0.225 |
| Heart Failure | 1999-2015 | -4.08 (-4.70 to -3.59) | P<.001 |
|  | 2015-2023 | 5.79 (4.41 to 7.68) | P<.001 |
| Ischemic Heart Disease | 1999-2003 | -0.38 (-3.01 to 5.43) | P=0.771 |
|  | 2003-2017 | -4.26 (-5.52 to -3.82) | P=0.014 |
|  | 2017-2023 | 4.26 (2.48 to 7.07) | P=0.005 |
| Liver Metastasis | 1999-2007 | -7.59 (-10.02 to -6.58) | P<.001 |
|  | 2007-2012 | -1.37 (-5.38 to 3.09) | P=0.372 |
|  | 2012-2016 | 8.48 (5.33 to 12.48) | P=0.010 |
|  | 2016-2023 | 2.62 (0.90 to 3.63) | P=0.023 |
| Lung Metastasis | 1999-2008 | -3.67 (-4.56 to -3.10) | P<.001 |
|  | 2008-2013 | 0.96 (-1.18 to 2.92) | P=0.312 |
|  | 2013-2016 | 10.37 (7.37 to 12.00) | P<.001 |
|  | 2016-2023 | 2.67 (1.94 to 3.24) | P<.001 |
| Pulmonary Embolism | 1999-2013 | -0.69 (-4.65 to 0.73) | P=0.336 |
|  | 2013-2023 | 4.14 (2.27 to 10.39) | P<.001 |
| Sepsis | 1999-2013 | -1.54 (-1.80 to -1.29) | P=0.001 |
|  | 2013-2017 | 4.42 (3.08 to 6.59) | P=0.005 |
|  | 2017-2020 | -2.02 (-3.26 to -0.13) | P=0.036 |
|  | 2020-2023 | 4.59 (3.03 to 7.64) | P<.001 |
| **West** | | | |
| Colorectal Cancer | 1999-2018 | -2.26 (-2.46 to -2.11) | P<.001 |
|  | 2018-2023 | 0.23 (-0.81 to 2.82) | P=0.560 |
| Heart Failure | 1999-2015 | -3.16 (-4.39 to -2.37) | P<.001 |
|  | 2015-2023 | 2.75 (0.62 to 7.13) | P=0.012 |
| Ischemic Heart Disease | 1999-2016 | -4.03 (-5.16 to -3.54) | P<.001 |
|  | 2016-2023 | 0.03 (-2.04 to 6.03) | P=0.915 |
| Liver Metastasis | 1999-2006 | -7.00 (-8.90 to -5.73) | P<.001 |
|  | 2006-2014 | 1.74 (-0.23 to 3.22) | P=0.076 |
|  | 2014-2017 | 11.16 (6.87 to 13.44) | P<.001 |
|  | 2017-2023 | 1.53 (-0.11 to 2.58) | P=0.063 |
| Lung Metastasis | 1999-2003 | -9.31 (-15.59 to -5.59) | P<.001 |
|  | 2003-2014 | 2.36 (0.89 to 3.47) | P=0.016 |
|  | 2014-2017 | 12.43 (7.27 to 14.91) | P<.001 |
|  | 2017-2023 | 0.40 (-1.73 to 1.67) | P=0.653 |
| Pulmonary Embolism | 1999-2019 | 0.53 (-1.85 to 1.23) | P=0.380 |
|  | 2019-2023 | 7.40 (1.85 to 18.16) | P=0.001 |
| Sepsis | 1999-2019 | -0.30 (-2.06 to 0.23) | P=0.210 |
|  | 2019-2023 | 4.77 (0.59 to 12.52) | P=0.010 |
| **Urbanization** | | | |
| **Large Metro** | | | |
| Colorectal Cancer | 1999-2012 | -3.01 (-3.34 to -2.86) | P<.001 |
|  | 2012-2020 | -2.18 (-2.50 to -1.32) | P=0.003 |
| Heart Failure | 1999-2001 | -9.13 (-12.99 to -3.44) | P=0.001 |
|  | 2001-2004 | 1.97 (-1.72 to 5.06) | P=0.232 |
|  | 2004-2009 | -7.36 (-10.62 to -5.75) | P=0.002 |
|  | 2009-2015 | -3.59 (-5.63 to 1.48) | P=0.109 |
|  | 2015-2020 | 3.59 (1.26 to 8.74) | P=0.020 |
| Ischemic Heart Disease | 1999-2006 | -3.90 (-4.73 to -2.30) | P=0.008 |
|  | 2006-2017 | -6.09 (-8.10 to -5.65) | P<.001 |
|  | 2017-2020 | -0.13 (-3.73 to 4.81) | P=0.963 |
| Liver Metastasis | 1999-2007 | -8.96 (-10.49 to -7.99) | P<.001 |
|  | 2007-2013 | -1.69 (-5.87 to 0.75) | P=0.137 |
|  | 2013-2016 | 11.05 (2.52 to 13.80) | P=0.036 |
|  | 2016-2020 | 2.75 (-0.98 to 5.24) | P=0.101 |
| Lung Metastasis | 1999-2006 | -4.87 (-9.75 to -3.07) | P=0.015 |
|  | 2006-2013 | -0.12 (-3.01 to 3.61) | P=0.964 |
|  | 2013-2020 | 5.37 (3.78 to 10.02) | P=0.008 |
| Pulmonary Embolism | 1999-2014 | -0.35 (-5.69 to 3.74) | P=0.297 |
|  | 2014-2020 | 2.43 (0.02 to 9.97) | P=0.049 |
| Sepsis | 1999-2002 | -4.31 (-9.19 to -0.18) | P=0.039 |
|  | 2002-2005 | 2.22 (-5.03 to 5.05) | P=0.315 |
|  | 2005-2011 | -3.55 (-7.02 to 2.90) | P=0.141 |
|  | 2011-2020 | 0.00 (-2.40 to 2.51) | P=0.897 |
| **Medium-Small Metro** | | | |
| Colorectal Cancer | 1999-2011 | -2.78 (-3.21 to -2.55) | P<.001 |
|  | 2011-2020 | -1.72 (-2.07 to -0.97) | P=0.009 |
| Heart Failure | 1999-2014 | -3.85 (-4.88 to -3.12) | P<.001 |
|  | 2014-2020 | 3.48 (0.77 to 11.20) | P=0.015 |
| Ischemic Heart Disease | 1999-2016 | -4.27 (-5.34 to -3.82) | P=0.002 |
|  | 2016-2020 | 1.29 (-2.73 to 9.71) | P=0.496 |
| Liver Metastasis | 1999-2007 | -7.69 (-9.73 to -6.68) | P<.001 |
|  | 2007-2012 | -1.26 (-5.54 to 2.82) | P=0.440 |
|  | 2012-2020 | 4.81 (3.74 to 7.47) | P=0.001 |
| Lung Metastasis | 1999-2007 | -3.39 (-5.44 to -2.78) | P=0.003 |
|  | 2007-2012 | -0.87 (-2.51 to 3.86) | P=0.342 |
|  | 2012-2017 | 8.06 (7.06 to 10.31) | P<.001 |
|  | 2017-2020 | -2.04 (-3.92 to 0.28) | P=0.065 |
| Pulmonary Embolism | 1999-2020 | 0.71 (-0.04 to 1.59) | P=0.066 |
| Sepsis | 1999-2020 | -0.46 (-0.87 to 0.02) | P=0.057 |
| **Nonmetropolitan** | | | |
| Colorectal Cancer | 1999-2006 | -2.47 (-4.01 to -1.95) | P<.001 |
|  | 2006-2020 | -1.48 (-1.67 to -0.91) | P=0.010 |
| Heart Failure | 1999-2013 | -4.68 (-7.01 to -3.53) | P<.001 |
|  | 2013-2020 | 3.20 (-0.27 to 14.37) | P=0.067 |
| Ischemic Heart Disease | 1999-2007 | -1.80 (-2.71 to -0.20) | P=0.036 |
|  | 2007-2012 | -7.08 (-10.74 to -5.03) | P<.001 |
|  | 2012-2020 | -0.91 (-2.00 to 0.98) | P=0.260 |
| Liver Metastasis | 1999-2010 | -6.32 (-7.28 to -5.59) | P<.001 |
|  | 2010-2020 | 5.45 (4.56 to 6.73) | P<.001 |
| Lung Metastasis | 1999-2010 | -3.35 (-5.66 to -1.91) | P<.001 |
|  | 2010-2020 | 4.79 (3.18 to 7.51) | P<.001 |
| Pulmonary Embolism | 1999-2020 | 0.30 (-0.52 to 1.24) | P=0.460 |
| Sepsis | 1999-2020 | 0.21 (-0.29 to 0.76) | P=0.378 |

**eTable 3. Pre-pandemic sensitivity analysis restricted to 1999-2019**

| **Characteristic** | **Temporal trend difference** | | **P value** |
| --- | --- | --- | --- |
|  | **Temporal segment** | **APC (95% CI), %** |  |
| **Sex** | | | |
| **Both** | | | |
| Heart Failure | 1999-2001 | -7.70 (-10.92 to -2.21) | P<.001 |
|  | 2001-2004 | -0.17 (-8.22 to 2.16) | P=0.716 |
|  | 2004-2012 | -6.00 (-7.90 to 2.27) | P=0.093 |
|  | 2012-2019 | 1.35 (-0.63 to 3.73) | P=0.081 |
| Ischemic Heart Disease | 1999-2006 | -3.25 (-3.79 to -2.49) | P<.001 |
|  | 2006-2016 | -5.53 (-6.35 to -5.19) | P<.001 |
|  | 2016-2019 | -0.42 (-2.70 to 3.19) | P=0.667 |
| Liver Metastasis | 1999-2008 | -7.89 (-8.84 to -7.20) | P<.001 |
|  | 2008-2013 | -0.22 (-7.18 to 2.11) | P=0.652 |
|  | 2013-2016 | 9.33 (-0.20 to 11.51) | P=0.054 |
|  | 2016-2019 | 2.74 (-1.40 to 5.66) | P=0.104 |
| Lung Metastasis | 1999-2008 | -3.79 (-4.57 to -3.22) | P=0.003 |
|  | 2008-2014 | 1.33 (-0.78 to 2.64) | P=0.119 |
|  | 2014-2017 | 9.04 (6.59 to 10.75) | P=0.004 |
|  | 2017-2019 | -0.09 (-2.67 to 2.96) | P=0.976 |
| Pulmonary Embolism | 1999-2004 | -1.93 (-6.67 to 0.09) | P=0.056 |
|  | 2004-2008 | 4.18 (1.58 to 7.78) | P=0.026 |
|  | 2008-2012 | -4.52 (-7.83 to -1.61) | P=0.026 |
|  | 2012-2019 | 3.19 (1.96 to 5.14) | P=0.011 |
| Sepsis | 1999-2013 | -1.61 (-2.68 to 0.45) | P=0.057 |
|  | 2013-2017 | 2.52 (-3.80 to 5.26) | P=0.193 |
|  | 2017-2019 | -5.11 (-9.62 to 0.74) | P=0.141 |
| Sepsis, 65-74 years | 1999-2013 | -2.04 (-3.91 to 3.31) | P=0.079 |
|  | 2013-2017 | 3.12 (-5.44 to 6.71) | P=0.242 |
|  | 2017-2019 | -6.17 (-12.49 to 1.15) | P=0.180 |

Abbreviations: APC, annual percent change; CI, confidence interval. The sensitivity analysis was restricted to 1999–2019 to minimize the potential influence of the COVID-19 pandemic. APC values were estimated using Joinpoint regression. Statistical significance was defined as a 2-sided P value of less than .05.
